# Ancestral and derived attributes of the *dlx *gene repertoire, cluster structure and expression patterns in an African cichlid fish

**DOI:** 10.1186/2041-9139-2-1

**Published:** 2011-01-04

**Authors:** Adina J Renz, Helen M Gunter, Jan MF Fischer, Huan Qiu, Axel Meyer, Shigehiro Kuraku

**Affiliations:** 1 Chair in Zoology and Evolutionary Biology, Department of Biology, University of Konstanz, Universitätsstrasse 10, 78457 Konstanz, Germany; 2 Zukunftskolleg, University of Konstanz, Universitätsstrasse 10, 78457 Konstanz, Germany; 3 Department of Pharmacology, Universitätsmedizin, Johannes Gutenberg University, Mainz, Obere Zahlbacher Strasse 67, 55101 Mainz, Germany

## Abstract

**Background:**

Cichlid fishes have undergone rapid, expansive evolutionary radiations that are manifested in the diversification of their trophic morphologies, tooth patterning and coloration. Understanding the molecular mechanisms that underlie the cichlids' unique patterns of evolution requires a thorough examination of genes that pattern the neural crest, from which these diverse phenotypes are derived. Among those genes, the homeobox-containing *Dlx *gene family is of particular interest since it is involved in the patterning of the brain, jaws and teeth.

**Results:**

In this study, we characterized the *dlx *genes of an African cichlid fish, *Astatotilapia burtoni*, to provide a baseline to later allow cross-species comparison within Cichlidae. We identified seven *dlx *paralogs (*dlx1a*, *-2a*, *-4a*, *-3b*, *-4b*, *-5a *and *-6a*), whose orthologies were validated with molecular phylogenetic trees. The intergenic regions of three *dlx *gene clusters (*dlx1a-2a*, *dlx3b-4b*, and *dlx5a-6a*) were amplified with long PCR. Intensive cross-species comparison revealed a number of conserved non-coding elements (CNEs) that are shared with other percomorph fishes. This analysis highlighted additional lineage-specific gains/losses of CNEs in different teleost fish lineages and a novel CNE that had previously not been identified. Our gene expression analyses revealed overlapping but distinct expression of *dlx *orthologs in the developing brain and pharyngeal arches. Notably, four of the seven *A. burtoni dlx *genes, *dlx2a*, *dlx3b*, *dlx4a *and *dlx5a*, were expressed in the developing pharyngeal teeth.

**Conclusion:**

This comparative study of the *dlx *genes of *A. burtoni *has deepened our knowledge of the diversity of the *Dlx *gene family, in terms of gene repertoire, expression patterns and non-coding elements. We have identified possible cichlid lineage-specific changes, including losses of a subset of *dlx *expression domains in the pharyngeal teeth, which will be the targets of future functional studies.

## Introduction

Cichlid fishes are amongst the premier models of evolution, as they have undergone rapid adaptive radiation to fill a multitude of ecological niches. This has been made possible, in part, by their striking diversity of jaw and tooth morphologies [1-5]. It has been hypothesized that one of the central factors that has permitted the cichlids' dramatic trophic radiation is their unique pharyngeal jaw apparatus, or 'throat jaws' [6,7]. These modified gill arches have taken on the role of food processing, freeing up the oral jaws for more specialized forms of food acquisition such as algal scraping [5]. An understanding of the genetic basis of cichlid craniofacial diversity first requires thorough dissections of the genes responsible for neural crest patterning. This knowledge can then be used to compare neural crest patterning between cichlid species, as this forms the basis for their highly variable oral and pharyngeal jaws [8-10].

Cichlid craniofacial specification and morphogenesis are beginning to be characterized at the molecular level. In the Nile tilapia, *Hox *paralog group 2 gene is expressed in the neural crest that populates the pharyngeal arches in a similar pattern to that of striped bass and zebrafish [11,12]. Later in development, numerous haplochromine cichlids express *Hox *genes in the dental mesenchyme directly surrounding the tooth germs in the lower pharyngeal jaw [13]. Albertson *et al*. identified *bmp4 *as a putative candidate for craniofacial diversity in a QTL study that compared cichlids with different jaw shapes [8]. Lastly, Kobayashi *et al*. identified *myofibril-associated glycoprotein 4 *gene (*magp4*) as a potentially important gene for cichlid craniofacial diversification as it is differentially expressed in the jaws of *Haplochromis chilotes *and *Haplochromis *sp."rockkribensis" [14].

The *Dlx *genes have a significant role in patterning the brain, jaw and teeth among amniotes [15], and represent excellent candidates to further characterize cichlid cranial morphogenesis. The *Dlx *genes are the vertebrate homologs of the *distal-less *(*dll*) gene first identified in *Drosophila*, which is known to be required for distal limb development [16]. *Dlx *and *dll *both belong to the homeobox-containing superfamily of transcription factors (reviewed in [15]). Six *Dlx *genes have been identified in mammals - they are likely to be derived from a tandem gene duplication event followed by chromosomal duplications [17]. This scheme also seems to hold in the leopard shark [18]. In contrast, in lampreys, although six *Dlx *genes have also been documented, their orthology relationships to the six *dlx *genes of gnathostomes (jawed vertebrates) are not fully resolved [19,20]. Previous studies have reported conserved non-coding elements in the *Dlx *clusters, suggesting that these intergenic elements contribute to the maintenance of syntenic relationships for sequential pairs of *dlx *genes within clusters [21-23].

Combinatorial expression patterns of mouse *Dlx *genes in the mandibular and hyoid arches were reported to be indispensable for establishing dorsoventral polarity between upper and lower jaw elements ('Dlx code') [24,25]. In the chicken, although *Dlx *gene expression fundamentally resembles that of mouse, *Dlx4 *was found to be pseudogenized [26]. In addition, the Dlx1-Dlx2 cluster has not been identified in its genome assembly even though the expression patterns of these individual genes have been reported [26,27]. In the zebrafish, it was shown that dorsoventral patterning of pharyngeal arches is achieved by regulatory genes including *dlx *genes [28-30]. This indicates that the Dlx-dependent patterning is derived from the common ancestor of at least all extant bony vertebrates. An analysis of a distantly-related teleost fish will provide clues that will help to reconstruct the ancestral state and infer secondary changes.

Among the teleost fishes, *dlx *gene functions have been well studied so far in zebrafish [28-31] and medaka [32]. Because of the teleost-specific genome duplication (TSGD), they possess more genes than tetrapods [33,34]. In cichlid fishes, only *dlx2 *(more precisely *dlx2a*) has been characterized so far [8,13]. In light of the nested expression patterns seen in the Dlx code [24,33], it is crucial to analyze the entire *dlx *gene repertoire of a single species. This is a challenge in species such as cichlid fishes where craniofacial morphology shows such unique features.

In this study, as a baseline to later explore the intra-Cichlidae variation, we provide comparative analysis of molecular phylogeny, genomic linkage and expression patterns of *dlx *genes between a selected cichlid species and non-cichlids. We identified seven *dlx *genes in an African cichlid fish, *Astatotilapia burtoni*, and analyzed their molecular phylogenies and their embryonic expression patterns. Intergenic regions of the three *dlx *gene clusters were also sequenced and subjected to searches for conserved non-coding elements (CNEs). Our intensive cross-species comparison revealed a relatively high level of conservation of CNEs among ray-finned fishes (Acanthopterygii) and additional lineage-specific gains/losses of CNEs amongst the teleost fishes as well as conservation of previously unidentified CNEs outside the *dlx *gene clusters. We detected differential expression of *dlx *genes particularly in the first and second pharyngeal arches, as previously characterized in zebrafish. In the developing pharyngeal teeth, we show that four of the seven identified *dlx *genes are expressed. Comparisons with other teleost fishes revealed possible losses of a subset of *dlx *expression in the pharyngeal teeth which to the best of our knowledge could be a unique feature of cichlid fishes.

## Results

### Identification of *A. burtoni dlx *genes

Through RT-PCR, we obtained sequences for seven *dlx *transcripts from *Astatotilapia burtoni*, each of which contained full-length protein-coding regions (see Materials and Methods). In the multiple alignment of the deduced amino acid sequences, all genes showed strong similarity to members of the *Dlx *subfamily of homeobox-containing genes, as they contain the characteristic amino acid residues of the dlx homeobox, TQTQVKIWFQN (Additional file 1). Based on sequence similarity to, and subsequent phylogenetic analyses with homologs from other teleost fishes, these seven cDNA sequences were inferred to be derived from orthologs of *dlx1a*, *-2a*, *-3b*, *-4a*, *-4b*, *-5a*, and *-6a*, and were designated accordingly. Although we performed *in silico *surveys of expressed sequence tags (ESTs) and partial genomic sequences from cichlid fishes currently available in NCBI dbEST http://www.ncbi.nlm.nih.gov/projects/dbEST/, no additional *Dlx *paralogs were found.

### Molecular phylogenetic analyses

**Figure 1:**
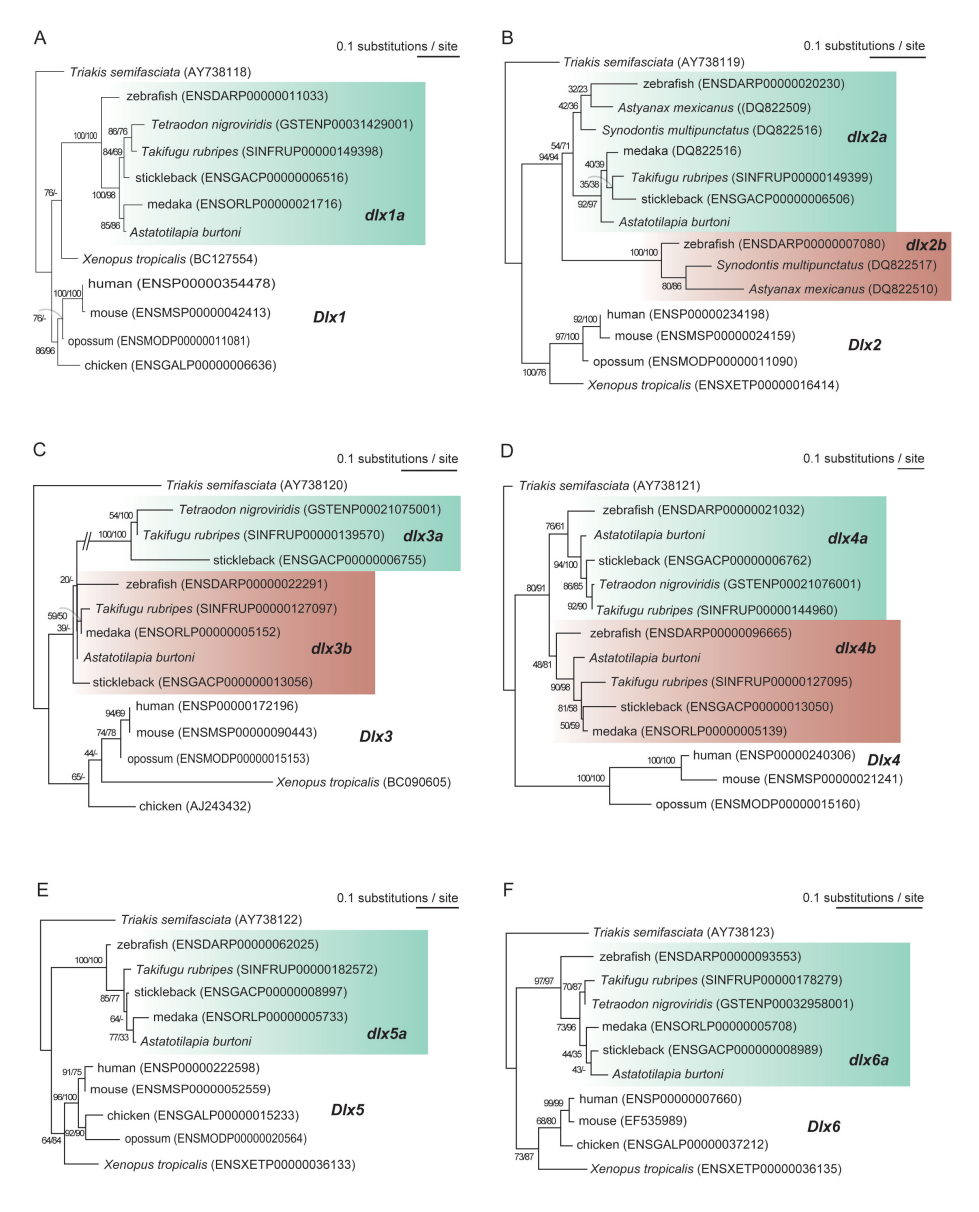
**Molecular phylogenetic trees including *Astatotilapia burtoni dlx *genes**. (A) *dlx1*. (B) *dlx2*. (C) *dlx3*. (D) *dlx4*. (E) *dlx5*. (F) *dlx6*. The trees were reconstructed with the maximum likelihood (ML) method (see Materials and methods). Bootstrap values were calculated with 100 resamplings. Support values at nodes indicate in order bootstrap probabilities in the ML and the NJ analyses. '-' indicates that the phylogenetic relationship supported by the ML was not reconstructed by the NJ method. In C, we do not show bootstrap probabilities for some nodes due to space limitations: 35 (ML) and 42 (NJ) for the node combining zebrafish *dlx3b *with its *T. rubripes *and medaka orthologs; 43 (ML) and '-' (NJ method did not support this relationship) for the node combining *A. burtoni dlx3b *with its orthologs of the zebrafish, *T. rubripes*, and medaka. For each of A-F, the orthologs of the leopard shark (*Triakis semifasciata*) and tetrapods were used as outgroups. The number of amino acid sites used for tree inference was as follows: (A) 239 amino acid sites (aa) (shape parameter for gamma distribution α = 0.50); (B) 174 aa (α = 0.37); (C) 137 aa (α = 0.34); (D) 128 aa (α = 0.44); (E) 216 aa (α = 0.29) and (F) 184 aa (α = 0.26). Accession numbers for the entries in GenBank and Ensembl are indicated in parentheses.

To confirm the identities of the newly discovered *A. burtoni dlx *genes to orthologs of other vertebrates, we conducted phylogenetic analyses. It was previously shown that the splits between *Dlx1-6 *occurred before the divergence between the chondrichthyan and osteichthyan lineages [17,19]. Therefore, we constructed phylogenetic trees for individual jawed vertebrate paralogs (*Dlx1-6*) using cartilaginous fish orthologs (namely leopard shark *Triakis semifasciata Dlx1-6*; [18]) as outgroup (Figure 1). Our survey of available genomic contigs of elephant shark (*Callorhinchus milii; *[35]) detected at least one exon for each of the six paralogs (*Dlx1-6*), but none of the identified contigs were long enough to cover the entire region employed in our phylogenetic analysis. In the currently available elephant shark genome sequences, we could not detect any additional *Dlx *genes that might be unique to the cartilaginous fish lineage.

In our molecular phylogenetic trees, all *Dlx *genes except *Dlx4*, (for which mammalian members show extremely long branches [36]), exhibited a dichotomy between teleost fishes and tetrapods, supported strongly by both the neighbor-joining (NJ) and maximum-likelihood (ML) methods (Figure 1D). The newly identified *A. burtoni *genes always grouped with homologs of other acanthomorpha species (Figure 1), consistent with previous studies on the phylogenetic relationships of teleost fishes [37-40]. Overall, our phylogenetic trees supported their orthologies and accordingly these *A. burtoni dlx genes *were named *dlx1a*, *dlx2a*, *dlx3b*, *dlx4a*, *dlx4b*, *dlx5a*, and *dlx6a*.

We observed relatively long branches at the base of teleost fishes for *dlx1a, -5a *and *-6a *(Figure 1A, E, F), suggesting an elevation of evolutionary rates for these genes. Moreover, *dlx2b *and -*3a *exhibited markedly longer branch lengths compared with their paralogs generated in the TSGD [33] (Figure 1B, C). This phylogenetic analysis also highlighted possible lineage-specific losses of *dlx *genes. Second teleost duplicates, generated by the TSGD, which we tentatively call *dlx1b*, *dlx5b *and *dlx6b *in the hypothetical common ancestor, are absent from all teleost fish genome sequences currently available. The absence of these putative duplicates implies that they were lost following their duplication in the stem lineage leading to teleosts. We also noted that the absence of *dlx3a *appears to be unique to Cypriniformes including the zebrafish, and that the absence of *dlx2b *appears to be unique to the percomorphs including pufferfishes, stickleback, medaka and cichlids (Figure 1B, C). These lineage-specific gene losses were also proposed by a previous report [32]. In our extended study, the absence of an *A. burtoni dlx3a *ortholog as well as a medaka *dlx4a *ortholog suggests additional gene losses.

In addition to the *A. burtoni *genes, we identified EST sequences in NCBI, encoding *dlx *genes of diverse teleost fishes, covering, for example, Salmoniformes, Siluriformes and Characiformes, including representatives of teleosts without sequenced genomes. After assembly of these previously unidentified *dlx*-encoding ESTs, we constructed molecular phylogenetic trees including them (Additional file 2; see also Materials and methods). According to the data available so far, none of these lineages have retained the *dlx *paralogs that should have existed just after the TSGD (namely *dlx1b*, *-5b *and *-6b*). All previously identified teleost-specific paralogs (*dlx1a*, -*2a*, -*3a*, -*3b*, -*4a*, -*4b*, -*5a*, and -*6a*) were retained by representative species from Salmoniformes, while only four of these (*dlx2a*, *-2b, -3a*, and *-5a*), including information in GenBank (*dlx2a *and *-2b *of *Synodontis multipunctatus*), were found in species from Siluriformes (Additional file 2B, C, E). Taken together, even though taxon and sequence sampling is obviously not complete, the loss of *dlx3a *(as seen in the zebrafish) at most dates back to the split of Cypriniformes from other otocephalan lineages (for example, Characiformes and Siluriformes) (Additional file 2C). On the other hand, since the presence of *dlx2b *is confined to the otocephalan representatives (Cypriniformes, Siluriformes and Characiformes) (Additional file 2B), this suggests that its ortholog was lost only in the basal lineage of Percomorpha (including stickleback, pufferfishes and medaka) at the latest. This gene loss might date back to the origin of Euteleostei before the split of Salmoniformes from others.

### *dlx *clusters: detection of putative *cis*-regulatory elements

**Figure 2:**
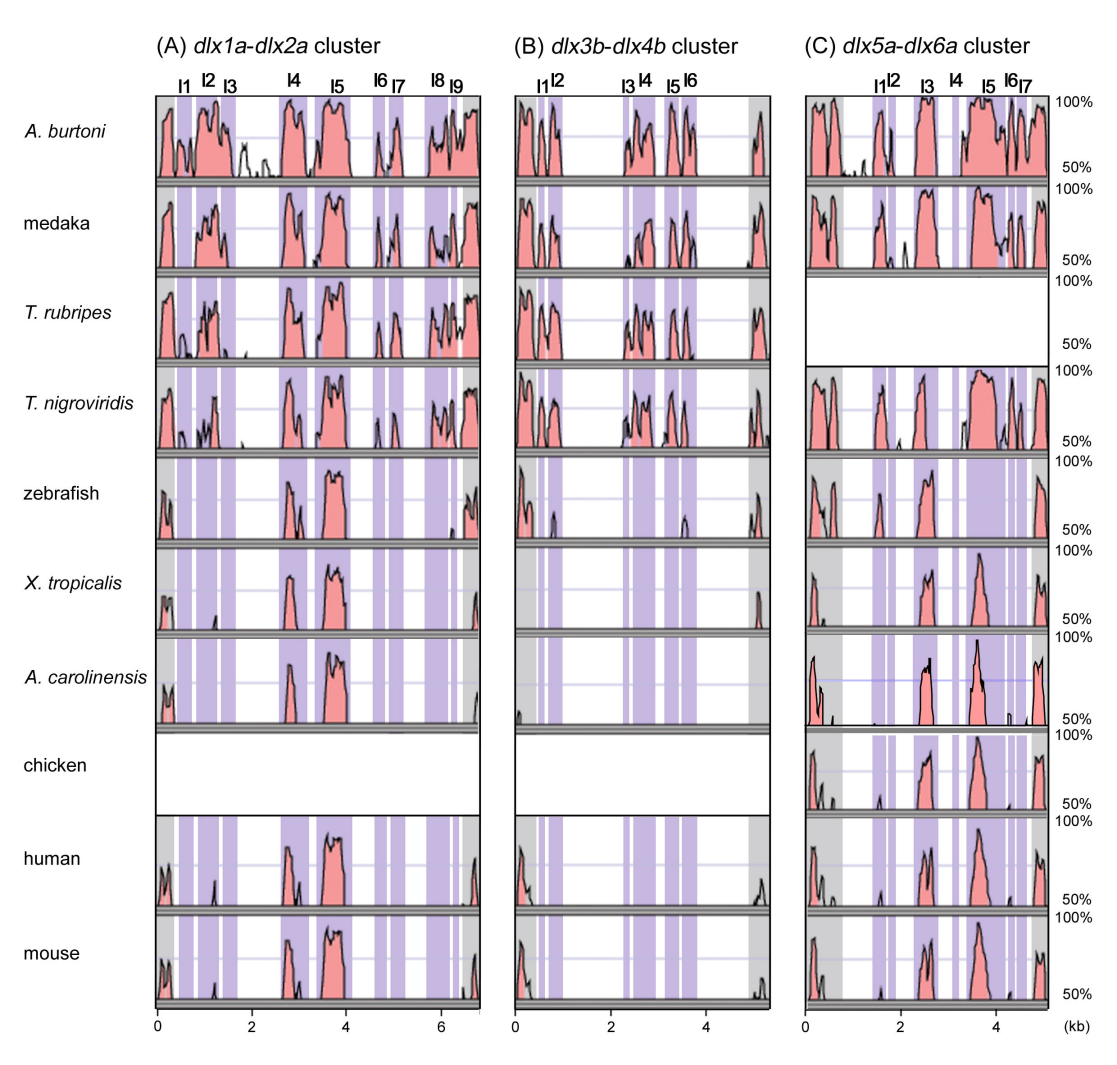
**Comparison of the intergenic regions of three *dlx *clusters among osteichthyans**. **(A) ***dlx1a*-*dlx2a *(*Dlx1*-*Dlx2*) cluster. **(B) ***dlx3b*-*dlx4b *(*Dlx3-Dlx4*) cluster. **(C) ***dlx5a*-*dlx6a *(*Dlx5-Dlx6*) cluster. Sequence similarity was visualized by mVista (see Materials and methods) using stickleback as a reference. Third exons of *dlx *genes are shown in grey and conserved non-coding elements (CNEs) in intergenic regions are shown in purple shading. Designation of detected CNEs, namely I12.1 to I12.9, I34.1 to I34.6 and I56.1 to I56.6, are shown at the top (see Materials and methods for our criterion for CNE annotation). See Additional file 3, for comparisons including the flanking regions of the *dlx *clusters.

In order to investigate patterns of conservation of potential *cis*-regulatory elements in intergenic portions of *dlx*-containing genomic regions, we conducted long PCR for *A. burtoni *between pairs of *dlx *genes whose orthologs in other teleost fishes form bi-gene clusters (see Materials and methods). With gene specific primers designed in the last exons of paired genes, genomic sequences for *A. burtoni dlx1a*-*2a*, *dlx3b*-*4b*, and *dlx5a*-*6a *clusters were successfully amplified. These sequences did not contain any non-*dlx *protein-coding region with strong similarity to sequences in public databases (also those without any strong similarity to sequences in other species; see Methods) and repetitive elements registered in the RepBase library other than simple repeats and low complexity sequences (see Materials and methods for details of gene prediction and repeat detection). Absence of any transcribed elements (for example, non-coding RNA) in the intergenic regions was also supported by Blastn searches using the entire intergenic sequences as queries against all available EST sequences, where we found no significant matches. The *A. burtoni *intergenic sequences were globally aligned and compared with their orthologous cluster sequences available for other vertebrates (Figure 2). In the intergenic regions of the *dlx1a*-*2a*, *dlx3b*-*4b*, and *dlx5a*-*6a *clusters, by applying the criterion of 70% identity within a 100-bp stretch, we identified nine, six, and seven conserved non-coding elements (CNEs), respectively, designated I12.1 to I12.9, I34.1 to I34.6 and I56.1 to I56.7 (Figure 2; also see Materials and methods for this naming). Even though our *A. burtoni *genomic sequences are limited to intergenic regions, we also compared 5-kb flanking regions on both ends for *dlx1a*-*2a*, *dlx3b*-*4b*, and *dlx5a*-*6a *clusters without *A. burtoni*, and detected 10, 5, and 13 CNEs that met the aforementioned similarity criterion (Additional file 3A, B, C).

Throughout the three *A. burtoni dlx *clusters, we did not detect the loss of any CNEs shared by other species, with one exception: a CNE tentatively named I12.3b (see Materials and methods for this naming). Notably, we identified CNEs shared by only a subset of teleost fishes. Most of them (for example, I12.7, I34.1, I34.2, I34.4, I34.5, I34.6, I56.6a and I56.7) were shared only between *A. burtoni*, medaka, stickleback and pufferfishes, excluding zebrafish, consistent with their phylogenetic relationships [37] (Figure 2). This type of conservation pattern was also frequently observed for flanking regions (Additional file 3A, B, C). One lineage-specific CNE I12.3ab was conserved only in medaka, *A. burtoni *and stickleback (Figure 2). Our comparison also revealed conservation of the previously characterized CNEs, I12.4, I12.5, I56.3, and I56.4 (I12b, I12a, I56ii and I56i, respectively; [21]) in a wider range of species, including *A. burtoni*, medaka, *Tetraodon nigroviridis *and anole lizard *Anolis carolinensis *(Figure 2). We also detected losses of CNEs in the zebrafish lineage (for example, I56.5) (Figure 2). In the flanking region of the *dlx5a*-*dlx6a *cluster, we detected an uncharacterized CNE, F56.9, which is conserved among all analyzed species, except the anole lizard where the corresponding region is not completely sequenced (Additional file 3).

We performed Blastn searches, using the identified CNE sequences of *A. burtoni *(for those in the intergenic regions) and stickleback (for those in the flanking regions) as queries, in currently available sea lamprey *Petromyzon marinus *and elephant shark *Callorhinchus milii *genome assemblies. In an elephant shark genome sequence (AAVX01041446 in GenBank), we identified a match for only the CNE I12.5, which is conserved in all osteichthyans surveyed in the present study (Additional file 4;also see [41]). In *P. marinus*, we could not find any homologous sequences to CNEs that we identified in this study. This should be reexamined with the anticipated whole genome sequences of this species and other cyclostomes.

### *dlx *gene expression analysis

We performed *in situ *hybridization for both whole embryos and sections for all seven *A. burtoni dlx *genes at 5, 7, 8, 10, 13 and 18 days post fertilization (dpf) (Table 1). Later stages were not included in our study as the calcification of bones and teeth in these stages was not compatible with the paraffin-based sectioning protocol we employed. Additionally, examination of the oral teeth in embryos 18 dpf was not always possible using this sectioning technique, and thus we have chosen to focus only on expression in the pharyngeal teeth.

**Table 1:** Embryonic expression domains of A. burtoni dlx genes.

Gene	Brain		Pharyngeal arches			Teeth		**Fin fold**^**a**^	**Other**^**a**^
					
	5 dpf	7, 8 and 10 dpf	5 dpf	7 dpf	10 dpf	Oral	Pharyngeal		
*dlx1a*	++	++ (di)	++ (I and II) - (III and IV) + (V and VI)	++	++	-	- (7 dpf) - (10 dpf) - (13 dpf)	-	
*dlx2a*	++	++ (di)	++ (I and II) + (III and IV) ++ (V and VI)	+	++	N.I.	+ (7 dpf) + (8 dpf) - (10 dpf)	-	+ (ov)
*dlx4a*	-	-	++ (I-VI)	++	++	+ (18 dpf)	++ (7 dpf) + (8 dpf) ++ (10 dpf) ++ (13 dpf)	-	
*dlx3b*	-	++ (olf)	++ (I-VI)	++	++	+ (18 dpf)	++ (7 dpf) ++ (8 dpf) ++ (10 dpf) ++ (13 dpf)	+ (cf) + (df) + (af)	++ (csm) + (ov)
*dlx4b*	-	++ (olf)	+ (I-VI)	++	++	-	- (7 dpf) - (10 dpf) - (13 dpf)	+ (cf)	+ (csm)
*dlx5a*	++	++ (di)	++ (I-VI)	++	++	+ (18 dpf)	++ (7 dpf) ++ (8 dpf) ++ (10 dpf)	+ (cf) + (df) + (af) + (pf)	+ (ov)
*dlx6a*	+	++ (di)	+ (I-VI)	-	+	N.I.	- (7 dpf) - (10 dpf)	-	

Expression domains with a high expression level were shown as '++', while '+' denotes a low expression signal. '-' indicates no expression signal detected and 'N.I.' indicates a case in which the expression pattern was not investigated. Additional spatial and temporal details of gene expression are shown in brackets. Abbreviations: di = diencephalon; cf = caudal fin; df = dorsal fin; af = anal fin; pf = pectoral fin; csm = circular smooth muscles of pharynx; olf = olfactory placode; ov = otic vesicle. ^a^Some of these expression domains are shown in Additional file 5 and Additional file 6, respectively.

### Brain

**Figure 3:**
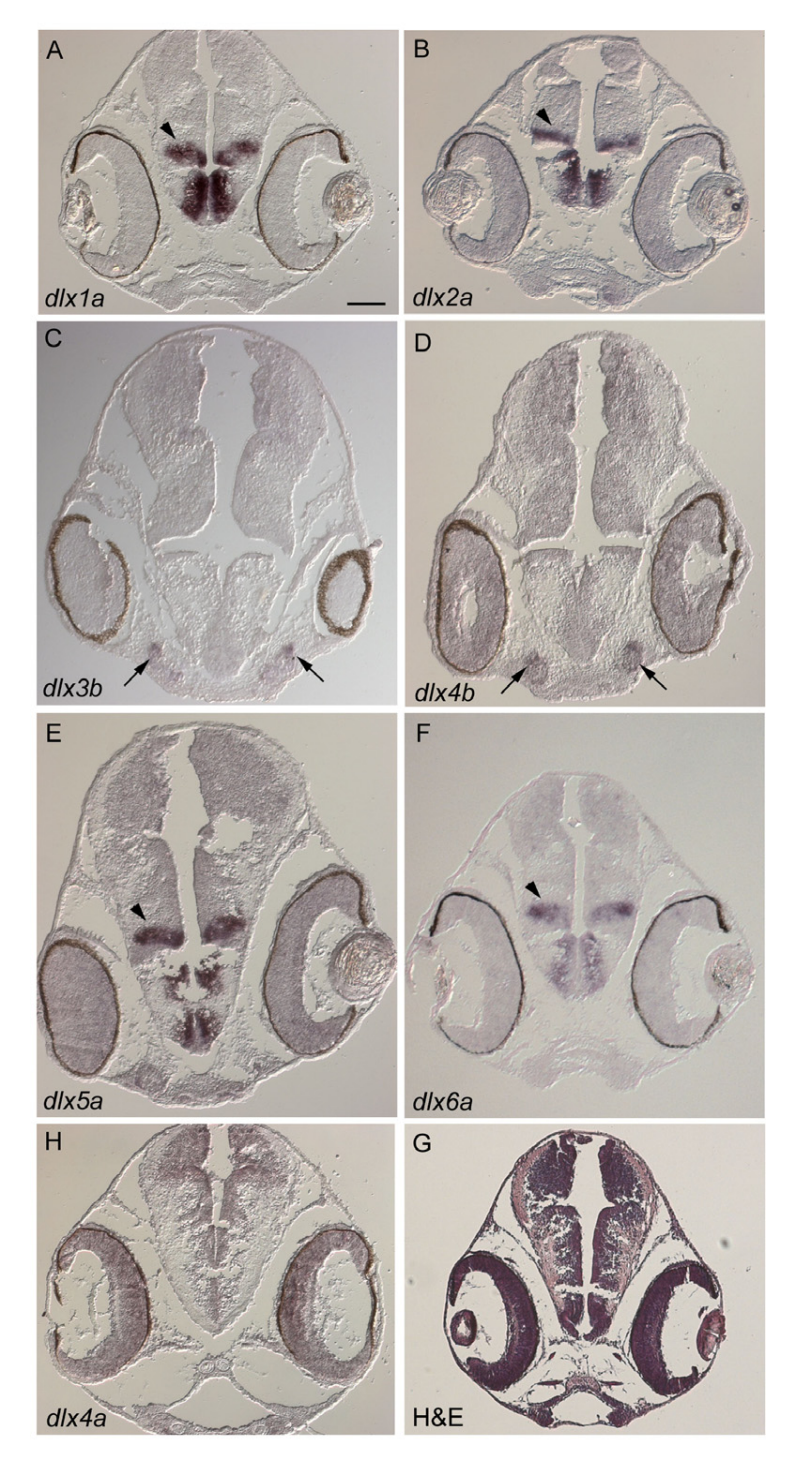
***A. burtoni dlx *expression patterns in the brain**. *In situ *hybridization on transverse sections at 7 dpf **(A to G)**. **(H) **Hematoxylin-eosin staining. *dlx1a *(A), *dlx2a *(B), *dlx5a *(E) and *dlx6a *(F) show similar expression patterns in the diencephalon of the forebrain (arrowheads). *dlx3b *(C) and *dlx4b *(D) also share expression signals in the region where the olfactory placodes are developing (arrows). No expression is seen for *dlx4a *(G). Scale bar in A: 100 μm. Anteroposterior levels of these photos are indicated in Additional file 9. Note that pigmentation persists in the eye.

**Figure 4:**
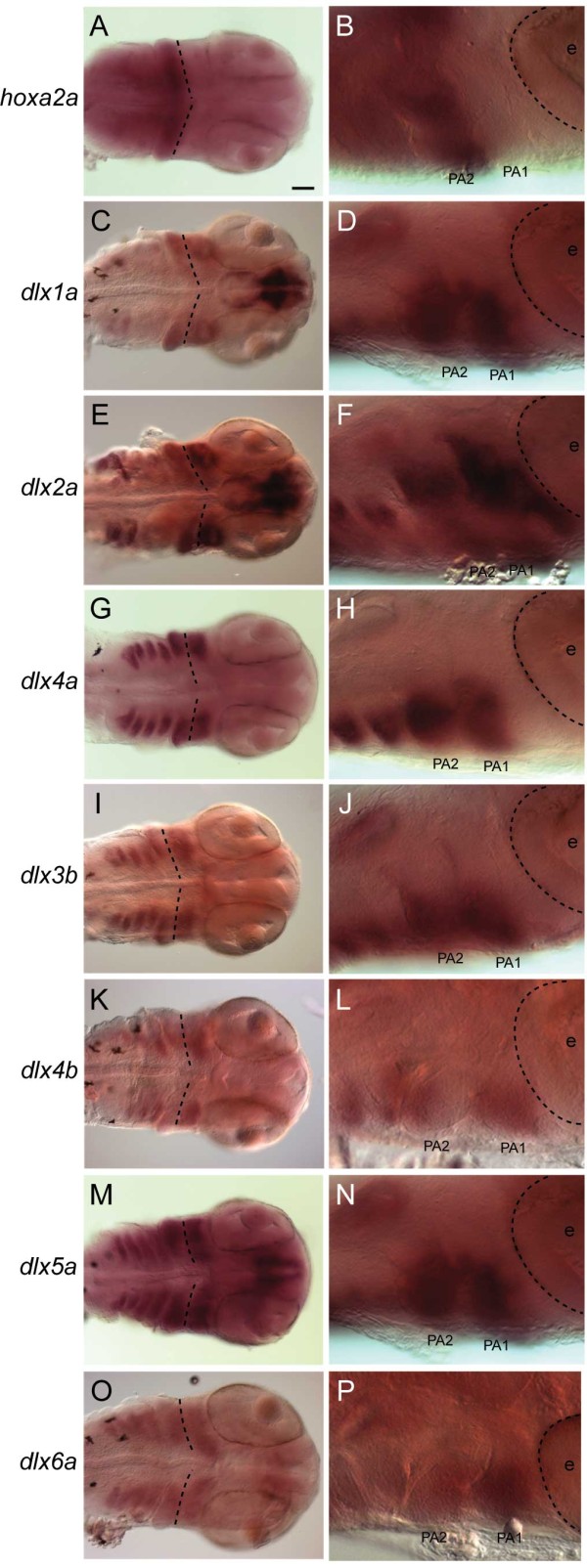
***A. burtoni dlx *expression patterns in the pharyngeal arches**. Whole-mount *in situ *hybridization at 5 dpf. Gene names are shown on the left. **(A, C, E, G, I, K, M, O) **Ventral view. The boundary between the PA1 and PA2, delineated by the anterior end of *hoxa2a *expression (A), is indicated with dotted lines. **(B, D, F, H, J, L, N, P) **Lateral view of the anterior pharyngeal arches. Anterior is to the right. The outline of the eye is indicated by a dotted line. In the PA1, along the DV axis, *dlx2a *expression extended more broadly than the other *dlx *genes (F), while *dlx3b *expression was more restricted (J). *dlx5a *and *dlx6a *expression extends more towards the ventral midline (M, O), while *dlx4a *is downregulated in ventral parts of the anterior PAs (G). Relatively weak expression of *dlx1a *and *dlx2a *was detected in PA3 and PA4 (C, E). Abbreviations: e = eye; PA1 = first pharyngeal arch; PA2 = second pharyngeal arch. Scale bar: 100 μm.

Two pairs of clustered genes, namely *dlx1a *and *-2a*, (arrowheads in Figure 3A, B), and *dlx5a *and *-6a*, (arrowheads in Figure 3E, F), showed similar expression patterns in the diencephalon of the brain at 7 dpf. *dlx3b *and *dlx4b *were expressed more anteriorly on the ventral side, where the olfactory placodes are located (arrows in Figure 3C, D). For *dlx4a*, no clear signal was detected in comparable sites at the stages we investigated (Figure 3G). At 10 dpf, expression signals of *dlx1a*, *-2a*, *-5a *and *-6a *genes were still detected in the median of the diencephalon (data not shown). No expression signals of *dlx3b*, -*4a *and *-4b *were detected at this stage. Expression signals in the brain for *dlx1a*, *-2a*, *-5a *and *-6a *were also observed in five dpf embryos (Figure 4C, D, G, H).

### Pharyngeal arches

At 5 dpf, intense expression was detected in the ventrolateral region of the ectomesenchyme in the developing pharyngeal arches (PAs) for all seven *A. burtoni dlx *genes (Figure 4). In parallel, we performed whole-mount *in situ *hybridization of *hoxa2a *gene, which is known to be expressed in the second and more posterior arches [12,42,43]. In PA1, which is marked by the absence of *hoxa2a *expression (Figure 4A, B), we detected expression of all analyzed *dlx *genes (Figure 4C-P). Of these, along the dorsoventral (DV) axis, *dlx2a *expression is distributed more broadly within PA1 (Figure 4F), while expression of at least *dlx3b *was more restricted (Figure 4J). In addition, the ventral-most areas of the anterior pharyngeal arches are devoid of *dlx4a *expression (Figure 4G). In contrast, expression of *dlx5a *and *-6a *extended further towards the ventral midline than the other *dlx *genes (Figure 4M, O). In the anterior part of the PA1 immediately behind the eye, no expression of *dlx4a*, *-3b *and *-5a *was observed (Figure 4H, J, N). We observed weaker expression of *dlx1a *and *dlx2a *in PA3 and PA4 than in other PAs (Figure 4C, E). Expression signals of *dlx1a*, *-3b*, *-4a*, *-4b *and *-5a *persisted until 10 dpf (data not shown). At 7 and 10 dpf, we observed only weak expression signals for *dlx2a*, while no clear signal of *dlx6a *expression was detected (data not shown).

### Pharyngeal teeth

**Figure 5:**
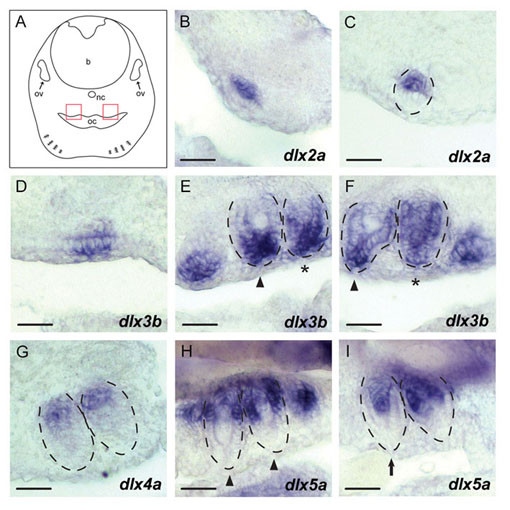
***A. burtoni dlx *expression patterns in pharyngeal tooth development**. Transverse sections were produced after performing whole mount *in situ *hybridization on 8 dpf *A. burtoni *embryos. Dorsal is to the top. **(A) **Schematic illustration of a transverse view. Red boxes indicate the regions of tooth development on the upper pharyngeal jaw magnified in **B-I**. Outlines of developing teeth were delineated with dotted lines based on hematoxilin-eosin staining performed after photographing these sections. (B, C) *dlx2a *expression. The signal in the dental mesenchyme was observed in a short period of early tooth development including morphogenesis stage (B, C). (D-F) *dlx3b *expression in the developing teeth. The signal was observed in the dental mesenchyme and oral epithelium during morphogenesis (D). The signal in the dental mesenchyme persisted until early differentiation (asterisks in E and F). In a later stage, the expression became more restricted towards the tip of the developing teeth (arrowheads in E and F). (G) *dlx4a *expression. The expression signal was observed at the base of the differentiating teeth in the dental mesenchyme and epithelium. (H, I) *dlx5a *expression. This gene was expressed more intensively in the dental epithelium than in the mesenchyme at the base of the differentiating teeth (arrowheads in H). Teeth in a later phase of differentiation showed intense mesenchymal expression signals compared with epithelial expression, which was restricted to the base of the inner dental epithelium (arrow in I). Developmental staging of teeth was based on Figure 4 of [32] for basic description in medaka and Figure 1 of [62] for that in a cichlid fish. Abbreviations: ov = otic vesicle; nc = notochord; b = brain; oc = oral cavity. Anteroposterior levels of these photos are indicated in Additional file 9. Scale bar: 20 μm.

Expression signals of *dlx2a*, *dlx3b*, *dlx4a *and *dlx5a *in the dentigerous area of the upper and lower pharyngeal jaws were first detected at 7 dpf, and persisted until 10 dpf (*dlx5a*) and 13 dpf (*dlx3b *and -*4a*), according to the stages we analyzed (Table 1). Of those, *dlx3b *expression in the teeth was the most intense throughout all developmental stages. At 8 dpf, *dlx3b *expression was observed in the dental mesenchyme and oral epithelium during morphogenesis (Figure 5D). The expression signal in dental mesenchyme persisted until early differentiation (asterisks in Figure 5E, F). In a later stage, the expression became more restricted towards the tip of developing teeth (arrowheads in Figure 5E, F). *dlx4a *expression was detected at 8 dpf at the base of the differentiating teeth in the dental mesenchyme and epithelium (Figure 5G). *dlx5a *showed a more dynamic pattern. At 8 dpf, this gene was expressed more intensively in the dental epithelium than in the mesenchyme at the base of the differentiating teeth (arrowheads in Figure 5H). Teeth in a later phase of differentiation showed intense mesenchymal expression signals compared with epithelial expression restricted to the base of the inner dental epithelium (arrow in Figure 5I). *dlx2a *expression in the dental mesenchyme was observed at 8 dpf in a short period of early tooth development including morphogenesis stage (Figure 5B, C). This signal was detected only in teeth positioned in the lateral extremities of the pharyngeal jaws. We did not detect expression of *dlx2a *in teeth at later stages of development. Throughout our analysis, we did not detect expression of *dlx1a*, *dlx4b *and *dlx6a *in developing teeth, even though these genes are expressed in other tissues in distinct, tissue-specific patterns (Figures 3 and 4).

### Other expression domains

We also detected *A. burtoni dlx *expression in other tissues. This includes expression of *dlx2a, -3b*, and -*5a *in the otic vesicle (Table 1). Intense signals of *dlx3b, dlx4b *and *dlx5a *were detected in the caudal fin rays at 7 dpf, 9 dpf and 10 dpf (Table 1). In the pectoral fin, we detected expression signals of *dlx5a *at 10 dpf (Additional file 5). Expression signals of *dlx3b *and *-4b *were also detected in the circular smooth muscles (csm) of the esophagus, posterior to the pharyngeal jaw, at 13 dpf (Additional file 6).

## Discussion

### The evolution of *dlx *gene repertoires in teleosts

The canonical genomic organization of the vertebrate *dlx *repertoire comprises three conserved bi-gene clusters [17]. One example violating this dogma is the pseudogenization of *Dlx4 *in chicken [26]. Based on our analysis, the anole lizard *Anolis carolinensis *retains an only partially annotated, but intact *Dlx4 *gene (Ensembl ID: ENSACAG00000005126), which is confirmed by the presence of its transcript in the NCBI EST Database (Accession ID: FG754683). We propose that the pseudogenization of the chicken *Dlx4 *might be a bird- or archosaurian lineage-specific event, and we can regard the '3 × 2 state' as the plesiomorphic condition of Dlx cluster architecture at least for jawed vertebrates. As observed previously for other genes [44], the identification of *dlx *genes in zebrafish [31] suggested an altered scheme for teleost fishes due to the additional whole genome duplication (TSGD, [33,34]). A more recent study on medaka *dlx *genes also produced a similar conclusion supporting the duplicated gene repertoires as well as its subsequent differentiation between teleost fish lineages [32].

**Figure 6:**
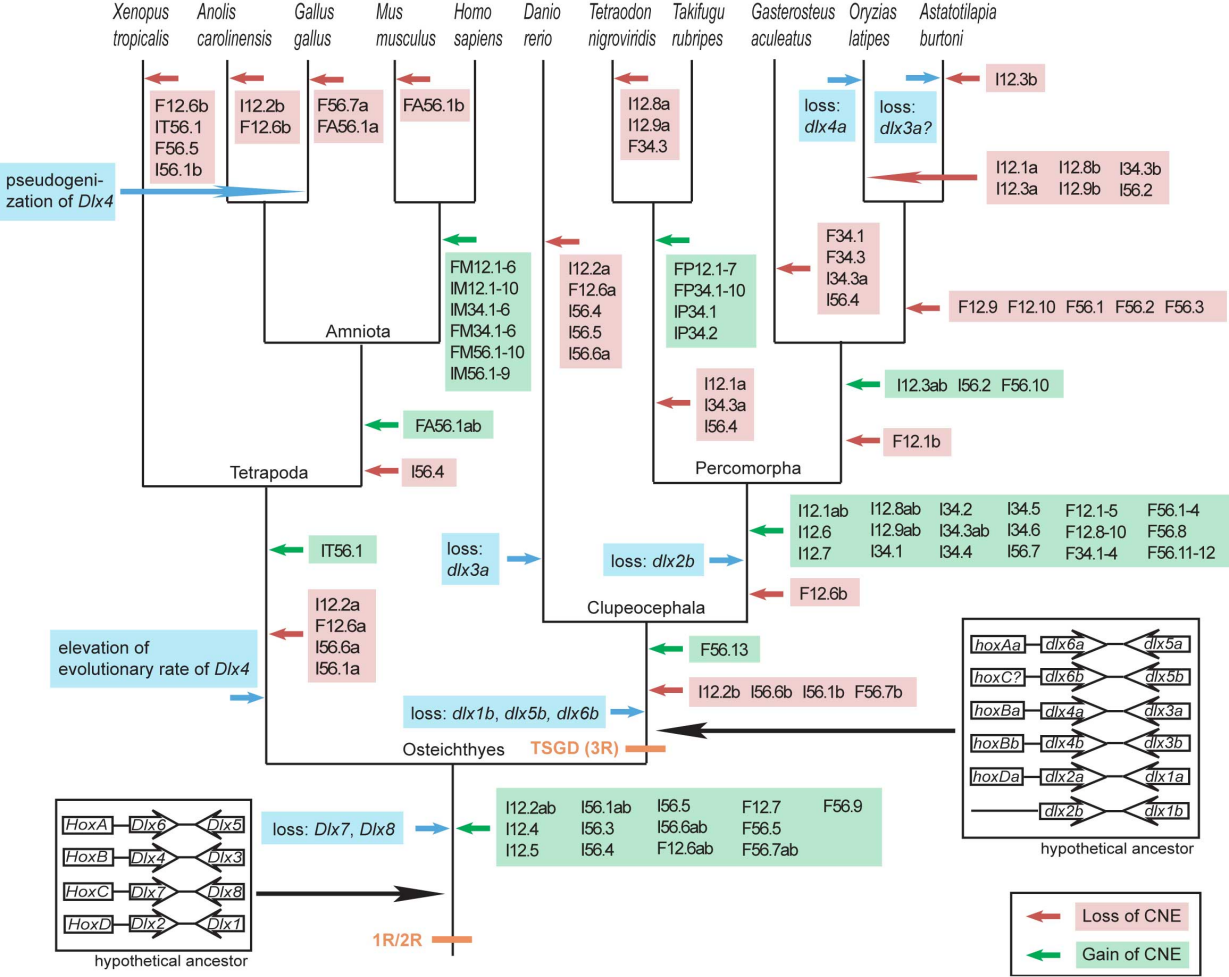
**A hypothesized scenario for *dlx *gene evolution**. Evolution of gene repertoires, gene cluster structure and conserved non-coding elements (CNEs) are shown (see Materials and Methods, for our criteria in assigning CNEs). Timing of gains and losses of CNEs is based on the most parsimonious interpretation. *Dlx7 *and *Dlx8 *are hypothetical genes that are thought to have existed immediately after two rounds of whole genome duplication. Alphabets in the names of CNEs: I = intergenic CNE; F = flanking CNE; M = mammal specific CNE; P = pufferfish specific CNE; A = amniote specific CNE; T = tetrapod specific CNE; 1R/2R = two rounds of whole genome duplications; TSGD (3R) = teleost-specific genome duplication.

Our identification of seven *A*. *burtoni dlx *genes provides evidence of further lineage-specific changes in *dlx *gene repertoires. The currently available medaka genome sequence does not contain the *dlx4a *gene, and our exhaustive search of its cDNA through RT-PCR with degenerate primers specifically designed for *dlx4a *failed as well. In contrast, the *A*. *burtoni dlx4a *gene was isolated with RT-PCR. However, *dlx3a*, which is present in the medaka genome and expressed during embryogenesis, was not detected by us in the *A. burtoni *transcriptome through RT-PCR with degenerate primers designed to specifically amplify *dlx3a*. Thus, neither medaka nor *A*. *burtoni *have been shown to possess an intact *dlx3a*-*dlx4a *cluster (Figure 6). Even though shown for other genes involved in vertebrate development [45,46], changes of developmental gene repertoires within Osteichthyes have not been intensively investigated so far. Our finding of possible differences in *dlx *gene repertoires within teleost fishes and even within Percomorpha suggests that more dynamic changes have occurred even in developmentally important regulatory genes between closely related taxa than was previously recognized. This observation on post-TSGD lineage-specific changes is paralleled by the situation of their *Hox *gene cluster complements that were also found to be different between different teleost fish lineages [44,47].

### Stepwise establishment of putative *cis*-regulatory elements

Our comparison of the intergenic regions of *dlx *clusters, including tetrapods, provided an overall picture of their putative *cis*-regulatory elements. Many gains of non-coding elements are deduced from the phylogenetic tracing even during the relatively recent evolution of teleost fishes (Figure 6). Additionally, we found a match of I12.5 in the elephant shark *C. milii*. This element has also been found in a BAC clone sequence containing *Dlx2 *[41], thus providing the first evidence of a putative *cis*-regulatory element in *Dlx *clusters outside Osteichthyes (Additional file 4). Our comparison also identified already functionally characterized CNEs in a wider range of vertebrates (see Results). At the same time, an element I12.3b, which has not been functionally characterized to date, was found to have been lost specifically in the cichlid lineage (Figure 6). In contrast, identification of cichlid-specific gains of CNE by means of phylogenetic footprinting [48] requires the addition of at least one more species that is sufficiently distant from *A. burtoni*.

The gains of the elements I12.3ab, I56.2 and F56.10 would support a closer phylogenetic relationship of stickleback to medaka/cichlid than to pufferfishes (Figure 6). The phylogenetic relationship among these percomorph fishes has not yet reached a consensus based on available molecular data [37,38,40,47]. These so-called 'rare genomic changes' [49] might serve as genomic synapomorphies for a stickleback-medaka-cichlid grouping.

### Dlx code in teleost fishes

*A. burtoni dlx *expression patterns in the brain closely resemble that of the mouse (Figure 3; [15]). Therefore, our riboprobes are regarded as specific enough to discern spatiotemporal regulation of *dlx *genes throughout embryogenesis. In light of the differential expression patterns of this group of genes observed in mouse and zebrafish ([24,28-30]; see also Introduction), we assessed dorsoventral (or proximodistal) distribution of *A. burtoni dlx *transcripts particularly in the PA1 and PA2. Our examination revealed the expression of all analyzed *A. burtoni dlx *genes in the PA1, the Hox-negative region, and in the *hoxa2a*-positive PA2 (Figure 4). At least *dlx2a *transcripts showed slightly wider distribution along the DV axis in the PA1 and PA2 than those of other *dlx *genes (Figure 4F). The broadest expression of *Dlx2 *homolog resembles the situation in zebrafish [28-30] and mouse [24], suggesting the conservation since the osteichthyan ancestor. Although not as distinct as the *dlx2a *expression, relatively broad expression of *dlx5a *along the DV axis is also described in zebrafish [28,30,31]. Our analysis on *A. burtoni dlx5a*, however, did not yield a similar pattern (Figure 4N). Including *dlx5a*, all the analyzed *dlx *genes except for *dlx2a *seemed to be expressed in a similar manner to each other in the PA1 and PA2 (Figure 4D, H, J, L, N and 4P), except that expression particularly of *dlx3*/*4 *genes did not protrude ventrally towards the midline (Figure 4G, I and 4K), as seen in zebrafish [31]. In summary, our analysis on *A. burtoni *supported the common ancestry of differential *dlx *expression patterns in the PA1/2 among teleost fishes, and also among osteichthyans. Equivalence of functional properties of Dlx code in cichlid fish could be tested by loss-of-function experiments as demonstrated in mouse and zebrafish [24,29].

### Possible roles for *dlx *genes in dentition: novelty in cichlids?

We performed an intensive analysis of *A. burtoni dlx *expression in pharyngeal teeth (Figure 5). Previously, *dlx *expression in teeth was described for zebrafish and medaka [31,32]. For cichlids, expression of *dlx2a *(designated '*Dlx2*' there) has been described in the teeth (oral, pharyngeal or both) for Lake Malawi species (*Tramitichromis intermedius*, *Labeotropheus fuelleborni*, *Dimidiochromis compressiceps *and *Metriaclima zebra *[8,13]). In *A. burtoni*, the *dlx2a *expression in the pharyngeal teeth was detected in a highly restricted manner both spatially and temporally (Figure 5B, C; see also Results). This restricted *dlx2a *expression was also documented in medaka and zebrafish [31,32]. *dlx5a *expression also exhibited striking dynamism in that its intense expression at the tooth base shifted from the dental epithelium to the mesenchyme in the course of tooth development (Figure 5H and 5I). As similar expression patterns are described in medaka and zebrafish [31,32], these dynamic expressions of *dlx2a *and *dlx5a*, as well as the most intense expression of *dlx3b *(see Results; [31,32]), are recognized as conserved features shared among the major lineages of Teleostei. *dlx3a*, which we could not identify in *A. burtoni*, has not been observed in the pharyngeal teeth in medaka [32]. *dlx4a*, the other gene on the same ancient bi-gene cluster as *dlx3a*, has not been identified in medaka [32]. Interestingly, in *A. burtoni*, we identified a *dlx4a *ortholog, and detected its relatively weak expression in pharyngeal teeth (Figure 5G), as well as in zebrafish [31]. We did not detect *dlx1a*, *dlx4b*, and *dlx6a *expressions in the pharyngeal teeth in the developmental stages we analyzed, while we detected expression signals of these genes in other tissues (Table 1). Of these, the absence of *dlx1a *expression in the pharyngeal teeth is documented also in medaka, while they express *dlx4b *and *dlx6a *[32]. Thus, it is possible that, in the lineage leading to cichlids after the split of the medaka lineage, pharyngeal tooth expression of *dlx4b *and *dlx6a *was secondarily lost. Further study would be required to determine whether the absence of those gene expressions is the result of our incomplete selection of stages or if they represent a genuine diversification of *dlx *functions after the split of the cichlid lineage from others. To confirm the cichlid lineage-specific changes, careful analyses of their expression in intermediate fish lineages (Pomacentridae and Embiotocidae; [10]) may also be necessary.

## Conclusion

Overall, inclusion of teleost fishes in the framework of vertebrate *Dlx *study and inclusion of a cichlid fish in that of teleost *dlx *study allowed us to highlight stepwise gains and losses of putative *cis*-regulatory elements as well as *dlx *family members at a higher resolution. Especially, in the lineage leading to an African cichlid *A. burtoni*, we discovered 1) a possible loss of *dlx3a *and 2) loss of the CNE I12.3b in the intergenic region of the *dlx1*-*dlx2 *cluster. We also detected possible loss of *dlx4b *and *dlx6a *expression in the developing pharyngeal teeth.

## Materials and methods

### Embryos

An inbred *Astatotilapia burtoni *line, originally provided by Hans Hoffmann (University of Texas, Austin, TX, USA), was kept at 24°C. Broods of up to 80 eggs were fertilized naturally and kept in the mothers' mouth until elicitation. For *in situ *hybridization, embryos were either fixed in 4% paraformaldehyde (PFA) in phosphate-buffered saline (PBS), dehydrated and stored in 100% methanol, or fixed in Serra's fixative (60% ethanol, 30% formaldehyde, 10% acetic acid) and kept in 100% ethanol at -20°C. The chorion and yolk were removed for embryos up to 6 dpf.

### Isolation and sequencing of cDNAs

Total RNA was extracted from whole embryos at 13 dpf using TRIzol reagent (Invitrogen, Carlsbad, CA, USA), and reverse transcribed into cDNA using a 3'RACE System (Invitrogen). The cDNA was used as a template in the following PCR amplification with the FastStart High Fidelity PCR System (Roche, Basel, Switzerland). Degenerate primers were designed based on the multiple alignment of major vertebrate *dlx *genes: 5'-GCG CAR ACN CAR GTN AAR ATH TGG TT-3' and 5'- GCG CAR GTN AAR ATH TGG TTY CAR AA-3' for the conserved amino acid stretches TQTQVKIWF and TQVKIWFQN, respectively. Nested 3'RACE PCRs were performed using the two degenerate primers, with universal primers that were designed to the 3' end of the oligo(dT) used for making cDNA. We amplified 3' cDNA fragments of *dlx3b*, *dlx5a*, *dlx4a *and *dlx4b *from this PCR. To isolate *dlx1a*, *dlx2a *and *dlx6a*, we designed forward gene specific primers based on partial exonic sequences of their putative orthologs in Malawi Lake cichlid fishes identified from genome shotgun reads available at Joint Genome Institute [50] (Additional file 7). These were used with the universal primers in 3'RACE reactions. 5' upstream sequences of the isolated cDNA fragments were amplified and sequenced with a 5'RACE System (Invitrogen). Details of PCR conditions and following procedures for cloning and sequencing were described previously [10]. cDNA sequences for *A. burtoni dlx *genes were deposited under accession numbers (EMBL: FN667596 to FN667602). The cDNA template for *hoxa2a *riboprobe was amplified using gene specific primers designed based on the *A. burtoni hoxa2a *genome sequence in GenBank (EF594313).

### Genomic DNA sequencing

Genomic DNA was extracted from a whole embryo of *A. burtoni *using the traditional phenol-chloroform protocol [51]. For long PCR against this genomic DNA, we designed primers specific to the 3' UTR of each isolated *dlx *gene. PCRs were performed using either a Long Range PCR System (Roche) or Long PCR Enzyme (Fermentas, St. Leon-Rot, Germany). Amplified DNA fragments were purified with the MinElute PCR Purification kit (Qiagen, Hilden, Germany), and cloned with the TOPO-XL Cloning kit (Invitrogen). Sequencing was performed through primer walking or random sequencing with EZ-Tn Kit (Epicentre, Madison, WI, USA). Genomic DNA sequences for *A. burtoni dlx *intergenic regions were deposited under accession numbers (EMBL: FN668537 to FN668539).

### Comparison of intergenic sequences

By using Genscan [52] and Augustus [53], we confirmed that there is no additional protein-coding sequence in the intergenic regions of the *A. burtoni dlx *clusters. Cross-specific comparison of intergenic sequences was performed using mVISTA [54] with the newly determined *A. burtoni *sequences as well as *dlx *clusters of model teleost fishes available in the Ensembl Genome Browser ([55]; version 54). Searches of the partial sea lamprey (version PMAR3) and elephant shark genome assemblies were performed using Blastn to detect local similarities, because no intact *Dlx *bi-gene cluster was contained in the genome assemblies. To detect CNEs, we were based on a criterion of 70% similarity in a 100 bp sequence stretch. See Additional file 8 for the naming of CNEs with 'a' and 'b' (for example, I12.3b). It should be noted that there could be functional *cis*-regulatory elements that are less similar or shorter than our criterion. A potential complication of pairwise comparisons arises when non-overlapping regions (tentatively named CNE a and CNE b in the present study) of a single CNE (CNE ab) are conserved between the query and each of the aligned sequences (Additional file 8).

### Molecular phylogenetic analyses

By using deduced amino acid sequences of the newly isolated sequences as queries, Blastp searches were performed to collect available homologous peptide sequences from Ensembl [55], and Genpept [56]. A multiple alignment was constructed using the alignment editor Xced where the alignment algorithm MAFFT is implemented [57]. Preliminary neighbor-joining trees were inferred on the Xced. Final trees were inferred using PhyML [58], assuming the JTT + I + Γ_4 _model. Bayesian posterior probabilities were calculated using MrBayes [59]. To confirm presence and absence of particular *dlx *genes in diverse teleost fish lineages, we ran an original Perl script to automatically detect orthologous sequences in assembled expressed sequence tags (ESTs) downloaded from NCBI dbEST [60]. Sequences found by this procedure were also included in molecular phylogenetic trees in Additional file 2.

### Whole-mount *in situ *hybridization

Digoxigenin (DIG)-labeled antisense and sense riboprobes were synthesized according to the manufacturer's instructions (Invitrogen) using SP6, T7 or T3 polymerase, according to the orientation of inserted template cDNA in the plasmid. After rehydration of fixed embryos and washing in PBTw (PBS containing 0.1% Tween 20), the embryos were post-fixed in 4% PFA/PBS for 20 minutes. Digestion with proteinase K (5 μg/ml, Roche) was adapted for cichlid embryos: 5 minutes at room temperature for embryos 5 dpf, 8 minutes for embryos 7, 8, 10 and 13 dpf and 10 minutes for embryos 18 dpf. After another post-fixation step, embryos were washed with DEPC-PBT, immersed in 50% formamide and prehybridized in hybridization buffer (50% formamide, 5× SSC, 1 mg/ml total yeast RNA, 50 μg/ml heparin, 0.1% Tween 20, in DEPC) for 3 h at 68°C. The specimens were then incubated in hybridization buffer with DIG-labeled RNA probes overnight at 68°C. After hybridization, the specimens were washed with 100% formamide solution (containing 50% formamide, 5× SSC, 0.1% Tween20 and 9 mM Citric acid in DEPC-water), 75%, 50% and 25% of this solution in 2% SSCTw (saline sodium citrate buffer containing 0.1% Tween 20) and in 2× SSCTw and 0.2× SSCTw at 68°C, each for 15 minutes. Then embryos were washed for each 10 minutes in 75%, 50% and 25% SSCTw in PBTw and finally in pure PBTw at room temperature. The specimens were soaked in 0.5% Blocking reagent (Roche) in PBTw for 30 minutes and subsequently the reagent was replaced by a 1:2000 dilution of anti-DIG-AP antibody in 0.5% Blocking solution and gently agitated at 4°C o/n. After incubation, the embryos were washed extensively in PBTw at room temperature, and hybridization was detected by incubation with NBT and BCIP (Roche). For histological observation in Figure 5, the stained embryos were dehydrated with a series of methanol, and embedded and sectioned as described below. Prepared sections were aligned on glass slides and deparaffinized for microscopic observation.

### Section *in situ *hybridization

Embryos were embedded in Paraplast (Carl Roth, Karlsruhe, Germany) and transverse sectioned with a microtome at a thickness of 8 μm. Detailed procedures were reported previously [61].

### Hematoxilin-eosin staining

Sectioned specimens were deparaffinized with three five-minute washes in xylene and were stepped into 70% ethanol. Staining in Mayer's Hematoxylin (Fluka, Buchs, Switzerland) was conducted for 20 minutes, and the slides were washed with running tap water for 5 to 10 minutes. Slides were incubated in 80% ethanol and stained with 0.25% EosinY (Fluka) in 80% ethanol for three to five minutes. They were quickly washed twice with 100% ethanol and twice in xylene. Sections were mounted with Eukitt (Fluka) for microscopic observation.

## Abbreviations

CNEs: conserved non-coding element; PA: pharyngeal arch; TSGD: teleost-specific genome duplication.

## Competing interests

The authors declare that they have no competing interests.

## Authors' contributions

SK and AM conceived the study. AJR conducted cDNA cloning, and JMFF sequenced intergenic genomic DNA. AJR and HQ performed molecular phylogenetic analyses. AJR and HMG collected embryos and analyzed gene expression patterns. SK wrote the first draft of the manuscript, and all authors contributed to the final version of the manuscript.

## Supplementary Material

Additional file 1 **Alignment of *dlx *genes and other homeodomain-containing genes**. Alignment shows *dlx1-6 *containing the homeodomain and flanking regions, in comparison to non-*dlx *relatives (yellow) of the Antennapedia (ANTP) class [63]. Teleost genes duplicated in the TSGD are shown in green (a paralog) and orange (b paralog). '#' represents amino acid residues conserved in the *dlx *gene family, while '*' represents amino acid residues conserved in all compared homeobox protein sequences. Click here for file

Additional file 2 **Molecular phylogenetic trees of *Dlx1-6 *including EST-derived teleost sequences**. (A) *Dlx1 *(207 amino acid sites (aa) employed in the analysis in total; shape parameter for gamma distribution α = 0.61). (B) *Dlx2 *(200 aa; α = 0.50). (C) *Dlx3 *(97 aa; α = 0.44). (D) *Dlx4 *(92 aa; α = 0.48). (E) *Dlx5 *(248 aa; α = 0.40). (F) *Dlx6 *(187 aa; α = 0.26). EST-derived sequences shown in bold are available upon request. Retention of *dlx1a *in *Pimephales promelas *and of *dlx4a *in *Oncorhynchus mykiss *was supported by analyses based on shorter alignments, but data are not shown here because of low confidence (data not shown). *Salmo salar *(Atlantic salmon), *Oncorhynchus mykiss *(rainbow trout), and *Osmerus mordax *(rainbow smelt) are categorized in the order Salmoniformes. *Ictalurus punctatus *(channel catfish) is categorized in Siluriformes. *Pimephales promelas *(fathead minnow) is categorized in Cypriniformes. Click here for file

Additional file 3 **Comparison of flanking genomic regions of *dlx *clusters**. **(A) ***dlx1a*-*dlx2a *cluster. **(B) ***dlx3b*-dlx*4b *cluster. **(C) ***dlx5a*-*dlx6a *cluster. Levels of sequence similarity were visualized by mVista (see Materials and methods) using stickleback as a reference. Exons are shown in gray shading. Conserved non-coding elements (CNEs) in intergenic regions and flanking regions are shown in purple and green shading, respectively. Designations of the detected CNEs, namely F12.1 to F12.10, F34.1 to F34.5 and F56.1 to F56.13, are shown at the top (see Materials and methods for our criterion CNE annotation). Note that *A. burtoni *sequences are not available for intronic and flanking regions. Note that the flanking region of the anole lizard *Dlx6 *gene contains a lot of 'N's, and this is mainly why many of the CNEs conserved between other species are absent. Click here for file

Additional file 4 **Nucleotide sequence alignments of two selected conserved non-coding elements (CNEs)**. **(A) **I12.5. **(B) **F56.9. Alignments were constructed by mVISTA. Sites with no substitutions are indicated with '+'. Click here for file

Additional file 5 ***dlx *expressions in *A. burtoni *fins**. Whole-mount *in situ *hybridization of *A. burtoni *embryos showing expression in the fin rays of the caudal fin for *dlx3b ***(A to C) **and *dlx5a ***(D to F) **at 7 dpf (A, D), 9 dpf (B, E) and 10 dpf (C, F), as well as in the pectoral fin for *dlx5a *at 10 dpf (G). Click here for file

Additional file 6 **Expression of *dlx *genes in the esophagus**. Section *in situ *hybridization in *A. burtoni *13 dpf **(B, C)**, as well as Hematoxylin-Eosin staining **(A)**. Strong signal of *dlx3b *transcripts (B) and lower signal of *dlx4b *transcripts (C) were detected in the circular smooth muscles (csm) surrounding the pharyngeal cavity posterior to the pharyngeal jaw. pc, pharyngeal cavity; ce, columnar epithelium. Arrows indicate expression. Scale bar: 100 μm. Anteroposterior planes of sectioning are indicated by shaded bars in a schematized *A. burtoni *embryo in Additional file 9. Click here for file

Additional file 7 **Oligonucleotide primers used to amplify *A. burtoni dlx *cDNAs**. Click here for file

Additional file 8 **Supporting illustration for CNE naming**. Naming scheme for CNEs with 'a', 'b' or 'ab'. Bars indicate putative *cis*-regulatory elements based on cross-species comparisons. In the pairwise comparisons between species 1 and 2 and species 1 and 3, we can detect similarities more than 70%, whereas the level of similarity for the pair of species 2 and 3 does not satisfy our criterion (100 bp and 70%). Accordingly we designate these similar regions (that overlap between species 1 and 2, and species 1 and 3) 'a' and 'b'. Click here for file

Additional file 9 **Anteroposterior levels of sections in a schematic *A. burtoni *embryo**. Anteroposterior planes of sectioning are indicated by shaded bars for the corresponding figures. Click here for file
