# Supplementary figures and images for: Ancestral and derived attributes of the dlx gene repertoire, cluster structure and expression patterns in an African cichlid fish
Source: EvoDevo. 2011 Jan 4;2:1. doi: 10.1186/2041-9139-2-1 (PMC3024246; doi:10.1186/2041-9139-2-1)

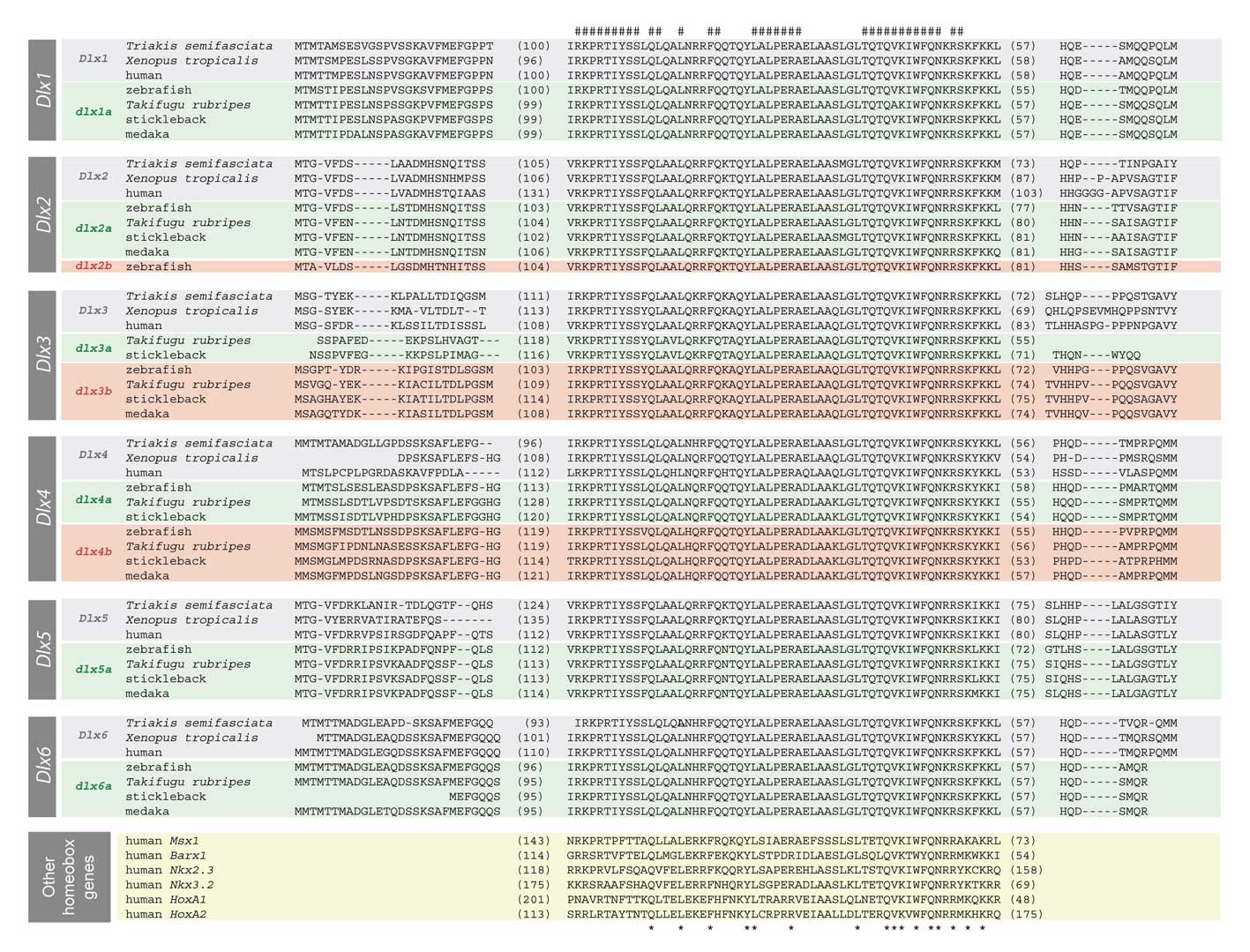

Supplement: Additional file 1 — Alignment of dlx genes and other homeodomain-containing genes. Alignment shows dlx1-6 containing the homeodomain and flanking regions, in comparison to non-dlx relatives (yellow) of the Antennapedia (ANTP) class [63]. Teleost genes duplicated in the TSGD are shown in green (a paralog) and orange (b paralog). '#' represents amino acid residues conserved in the dlx gene family, while '*' represents amino acid residues conserved in all compared homeobox protein sequences. [file 2041-9139-2-1-S1.JPEG]

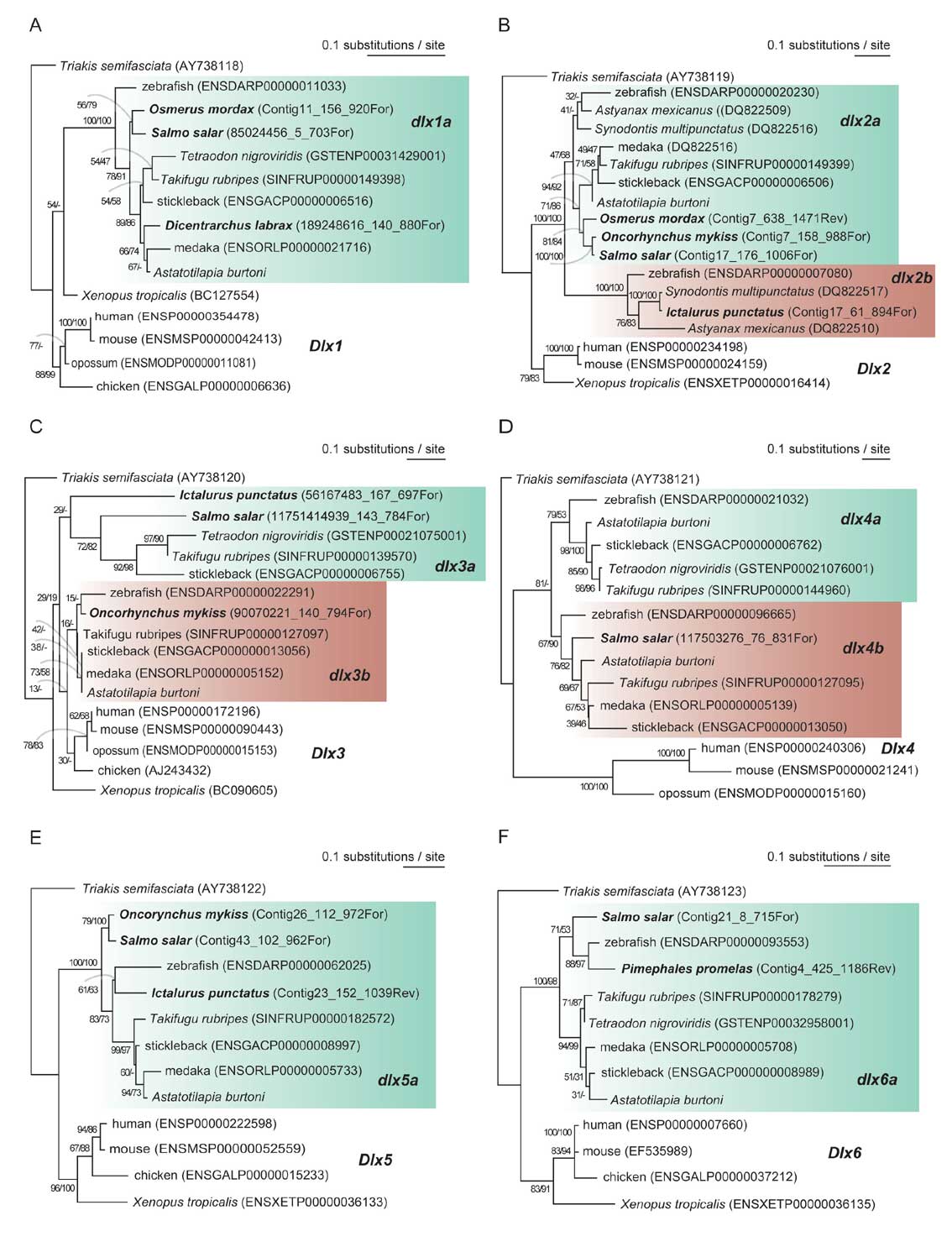

Supplement: Additional file 2 — Molecular phylogenetic trees of Dlx1-6 including EST-derived teleost sequences. (A) Dlx1 (207 amino acid sites (aa) employed in the analysis in total; shape parameter for gamma distribution α = 0.61). (B) Dlx2 (200 aa; α = 0.50). (C) Dlx3 (97 aa; α = 0.44). (D) Dlx4 (92 aa; α = 0.48). (E) Dlx5 (248 aa; α = 0.40). (F) Dlx6 (187 aa; α = 0.26). EST-derived sequences shown in bold are available upon request. Retention of dlx1a in Pimephales promelas and of dlx4a in Oncorhynchus mykiss was supported by analyses based on shorter alignments, but data are not shown here because of low confidence (data not shown). Salmo salar (Atlantic salmon), Oncorhynchus mykiss (rainbow trout), and Osmerus mordax (rainbow smelt) are categorized in the order Salmoniformes. Ictalurus punctatus (channel catfish) is categorized in Siluriformes. Pimephales promelas (fathead minnow) is categorized in Cypriniformes. [file 2041-9139-2-1-S2.JPEG]

**A**

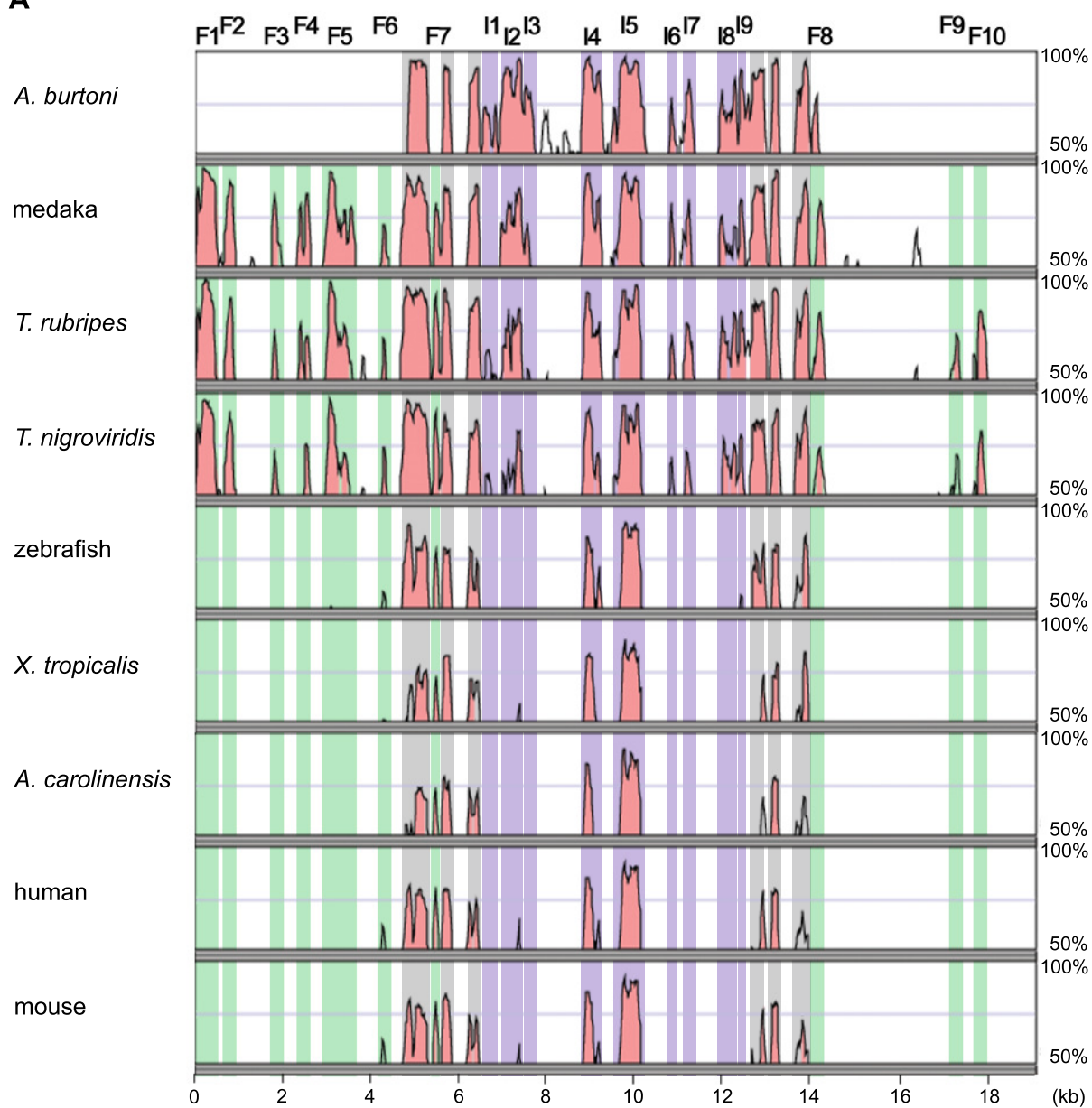

**B**

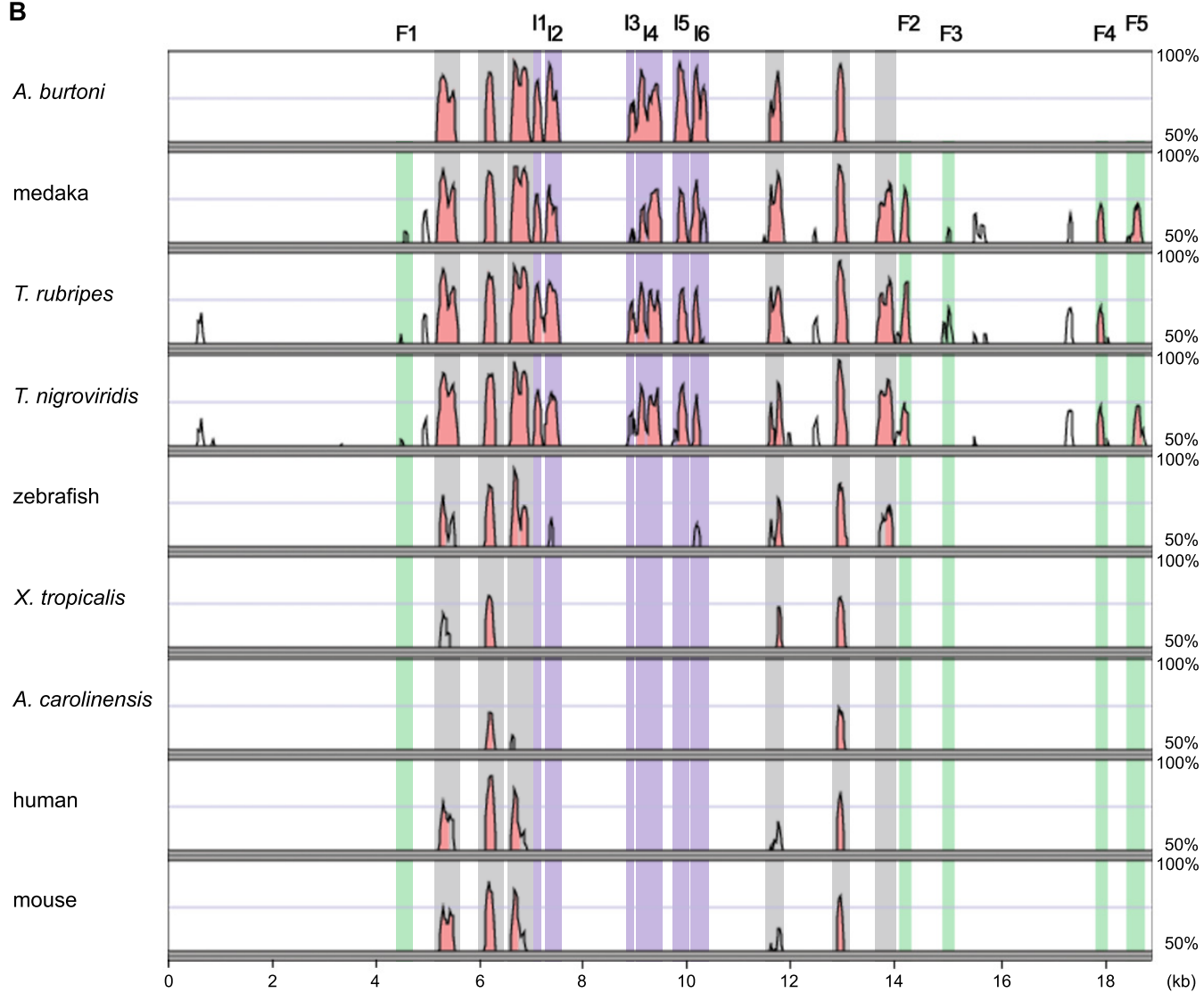

**C**

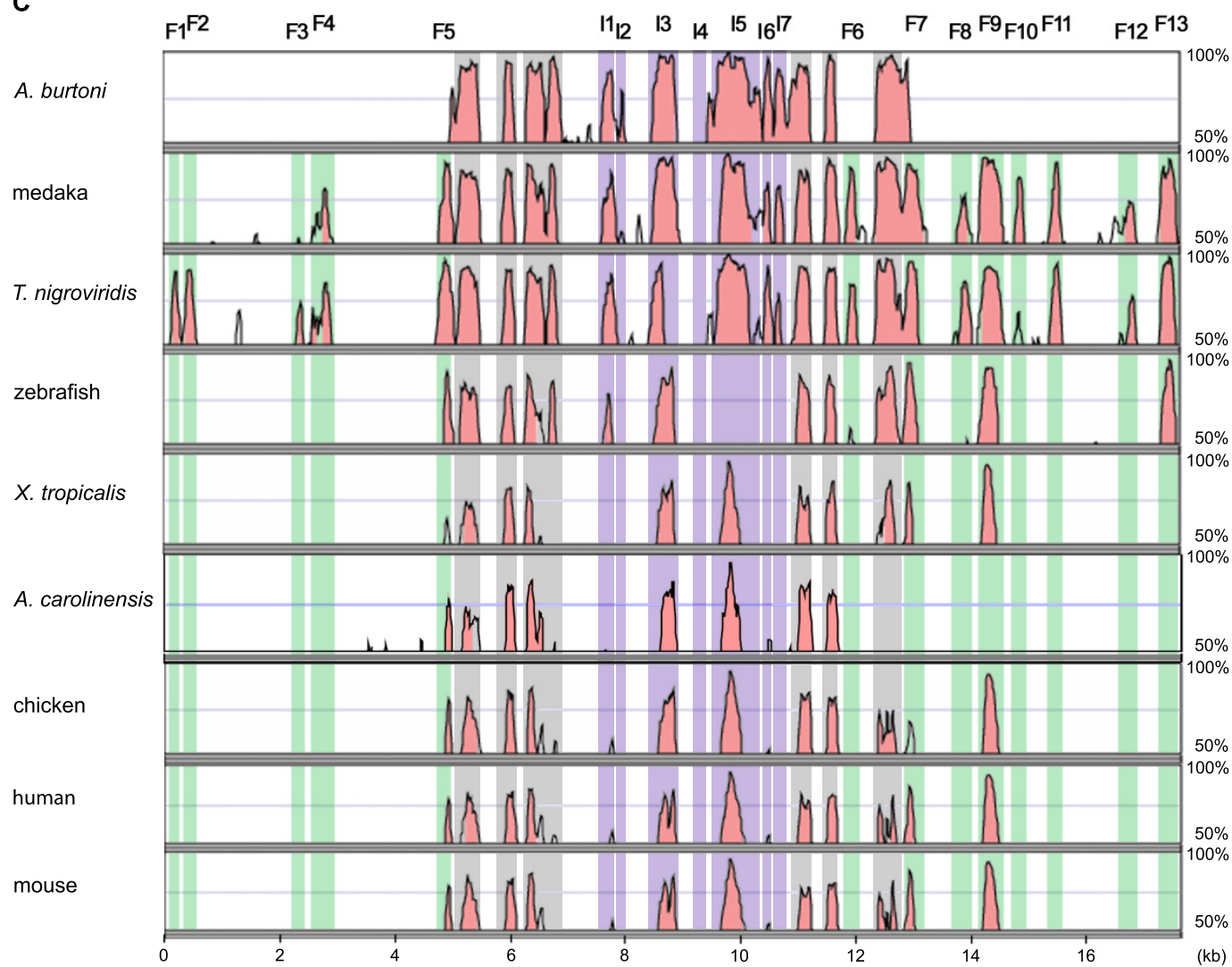

Supplement: Additional file 3 — Comparison of flanking genomic regions of dlx clusters. (A) dlx1a-dlx2a cluster. (B) dlx3b-dlx4b cluster. (C) dlx5a-dlx6a cluster. Levels of sequence similarity were visualized by mVista (see Materials and methods) using stickleback as a reference. Exons are shown in gray shading. Conserved non-coding elements (CNEs) in intergenic regions and flanking regions are shown in purple and green shading, respectively. Designations of the detected CNEs, namely F12.1 to F12.10, F34.1 to F34.5 and F56.1 to F56.13, are shown at the top (see Materials and methods for our criterion CNE annotation). Note that A. burtoni sequences are not available for intronic and flanking regions. Note that the flanking region of the anole lizard Dlx6 gene contains a lot of 'N's, and this is mainly why many of the CNEs conserved between other species are absent. [file 2041-9139-2-1-S3.PDF]

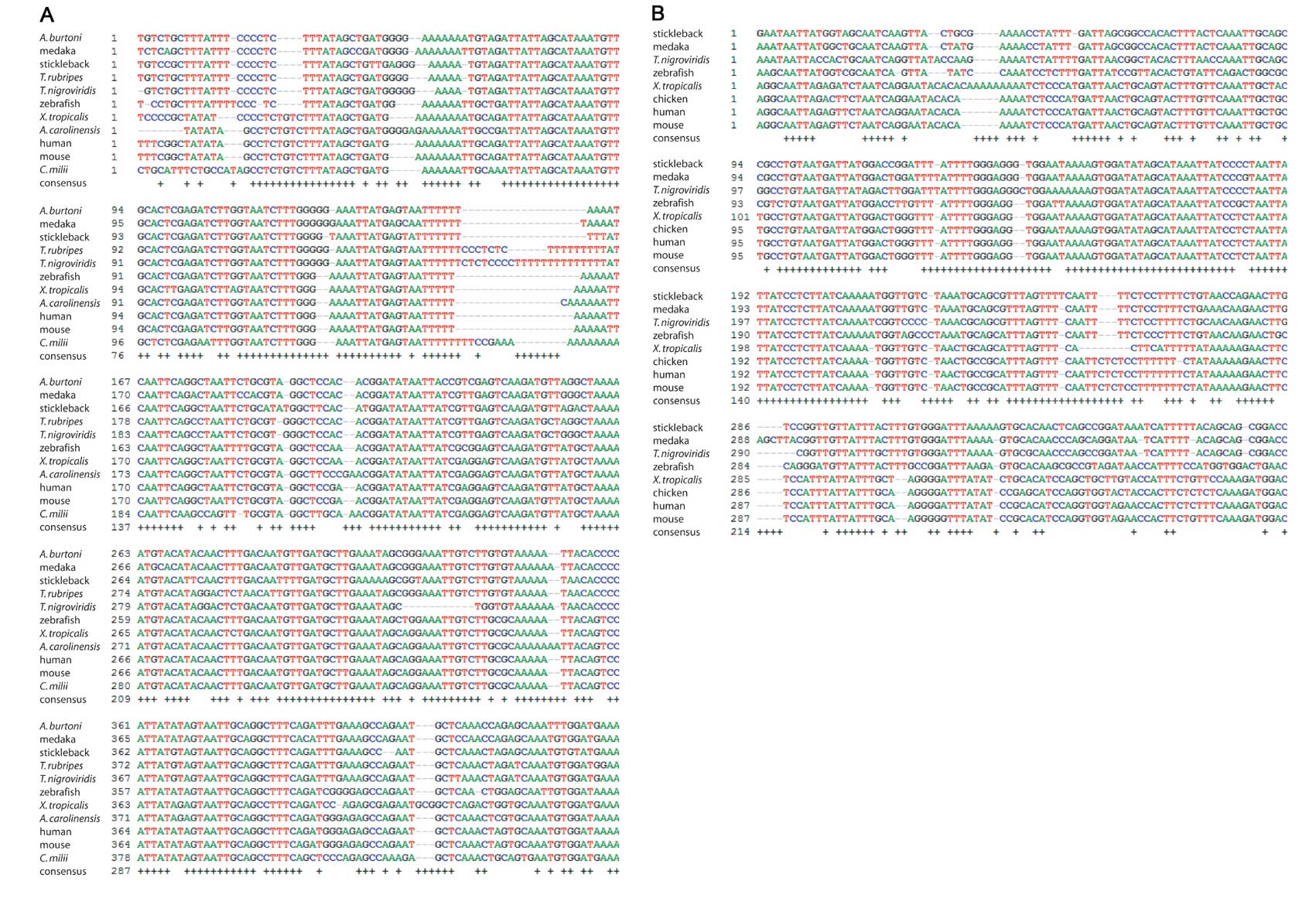

Supplement: Additional file 4 — Nucleotide sequence alignments of two selected conserved non-coding elements (CNEs). (A) I12.5. (B) F56.9. Alignments were constructed by mVISTA. Sites with no substitutions are indicated with '+'. [file 2041-9139-2-1-S4.JPEG]

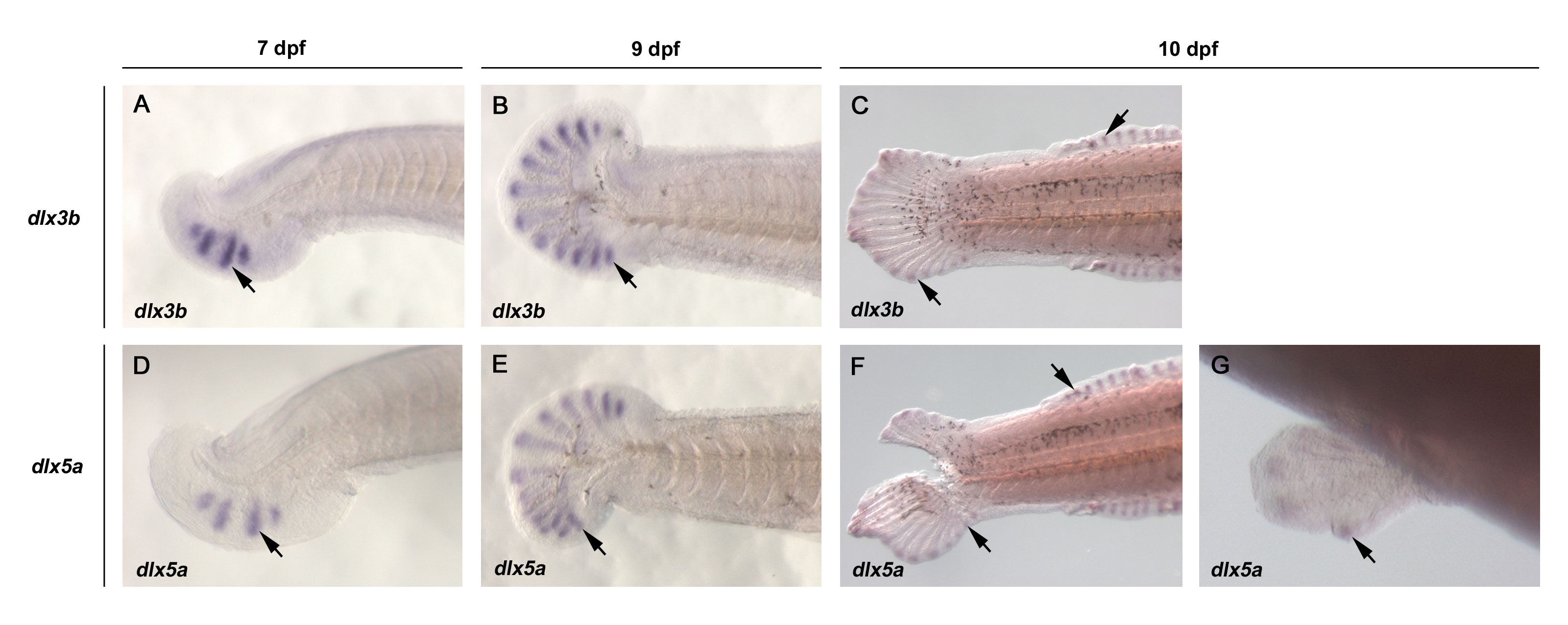

Supplement: Additional file 5 — dlx expressions in A. burtoni fins. Whole-mount in situ hybridization of A. burtoni embryos showing expression in the fin rays of the caudal fin for dlx3b (A to C) and dlx5a (D to F) at 7 dpf (A, D), 9 dpf (B, E) and 10 dpf (C, F), as well as in the pectoral fin for dlx5a at 10 dpf (G). [file 2041-9139-2-1-S5.JPEG]

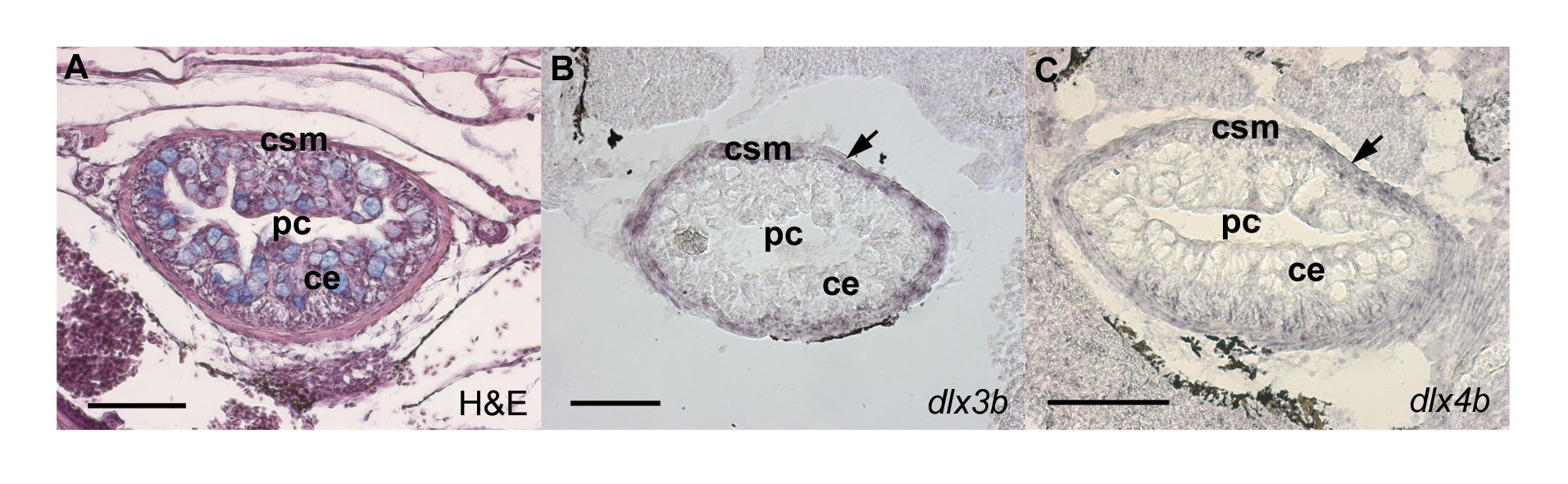

Supplement: Additional file 6 — Expression of dlx genes in the esophagus. Section in situ hybridization in A. burtoni 13 dpf (B, C), as well as Hematoxylin-Eosin staining (A). Strong signal of dlx3b transcripts (B) and lower signal of dlx4b transcripts (C) were detected in the circular smooth muscles (csm) surrounding the pharyngeal cavity posterior to the pharyngeal jaw. pc, pharyngeal cavity; ce, columnar epithelium. Arrows indicate expression. Scale bar: 100 μm. Anteroposterior planes of sectioning are indicated by shaded bars in a schematized A. burtoni embryo in Additional file 9. [file 2041-9139-2-1-S6.JPEG]

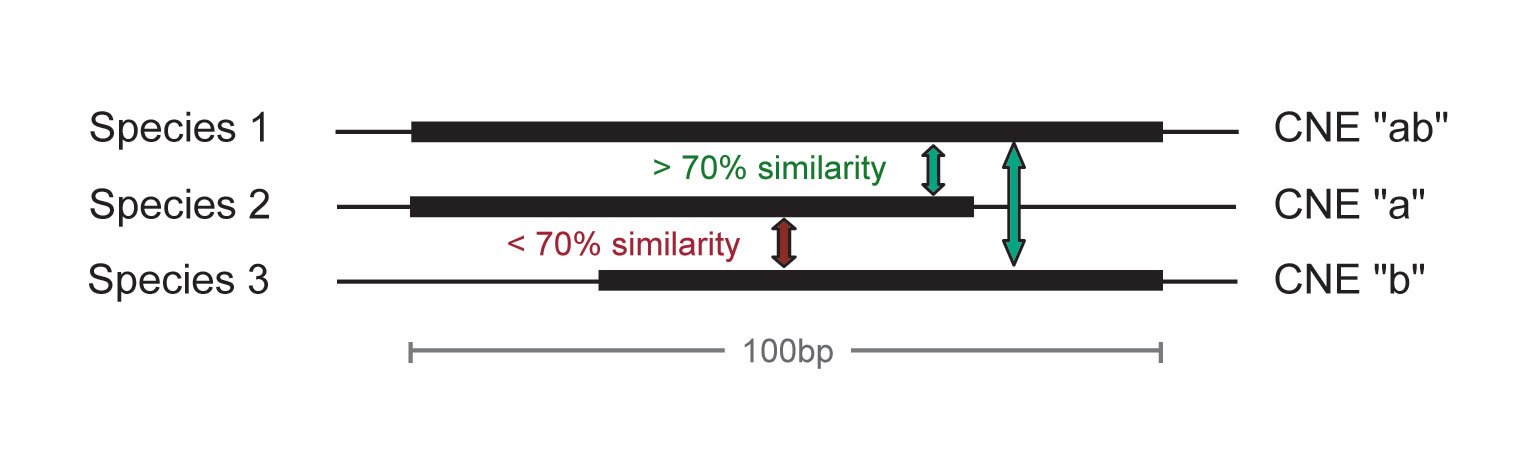

Supplement: Additional file 8 — Supporting illustration for CNE naming. Naming scheme for CNEs with 'a', 'b' or 'ab'. Bars indicate putative cis-regulatory elements based on cross-species comparisons. In the pairwise comparisons between species 1 and 2 and species 1 and 3, we can detect similarities more than 70%, whereas the level of similarity for the pair of species 2 and 3 does not satisfy our criterion (100 bp and 70%). Accordingly we designate these similar regions (that overlap between species 1 and 2, and species 1 and 3) 'a' and 'b'. [file 2041-9139-2-1-S8.JPEG]

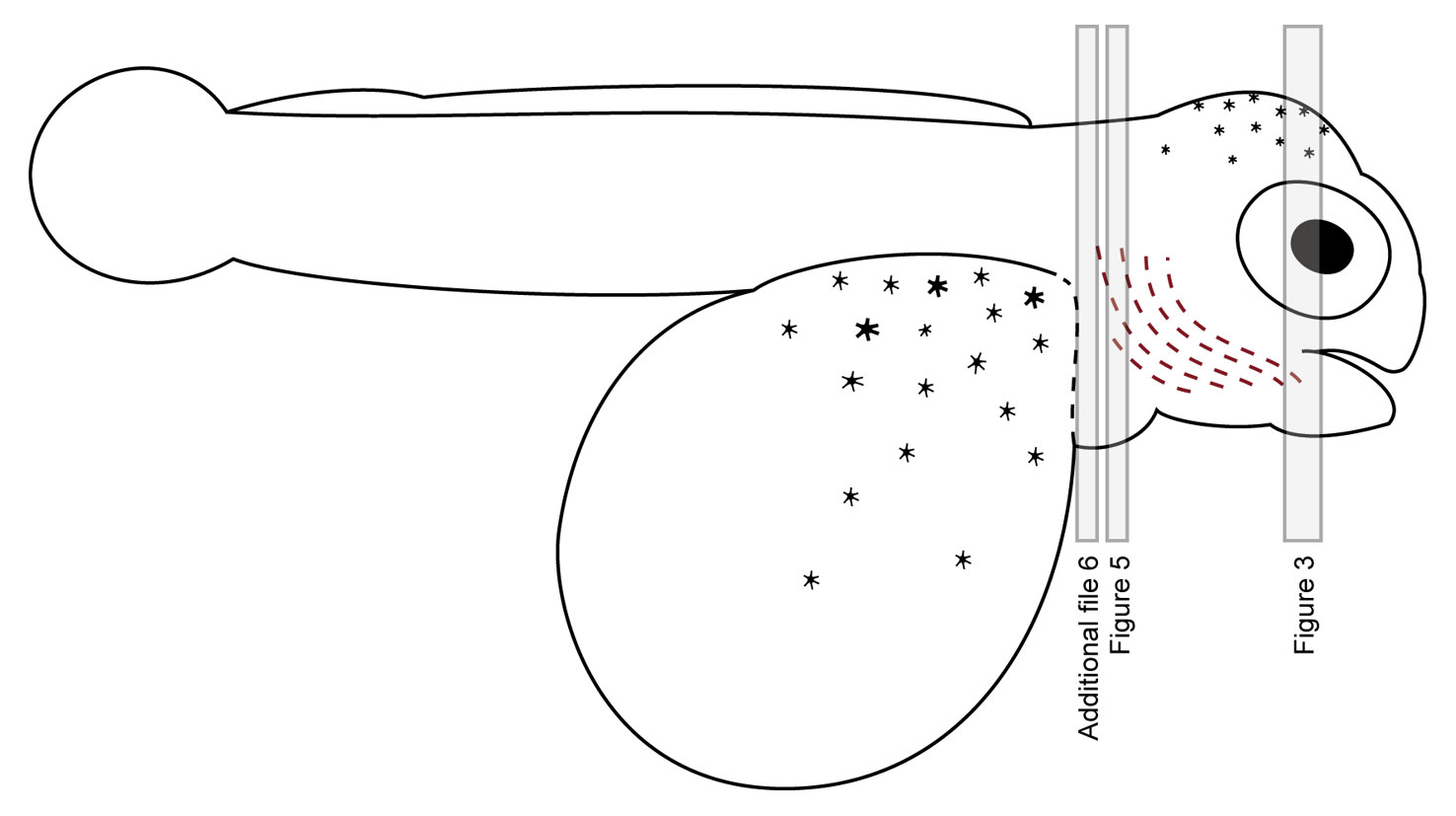

Supplement: Additional file 9 — Anteroposterior levels of sections in a schematic A. burtoni embryo. Anteroposterior planes of sectioning are indicated by shaded bars for the corresponding figures. [file 2041-9139-2-1-S9.JPEG]
