# Supplementary material for: Ancestral and derived attributes of the dlx gene repertoire, cluster structure and expression patterns in an African cichlid fish
Source: EvoDevo. 2011 Jan 4;2:1. doi: 10.1186/2041-9139-2-1 (PMC3024246; doi:10.1186/2041-9139-2-1)
Supplement: Additional file 7 — Oligonucleotide primers used to amplify A. burtoni dlx cDNAs. [file 2041-9139-2-1-S7.PDF]

Additional file 7. Oligonucleotide primers used to amplify *A. burtoni dlx* cDNAs.

Gene	Used in the first or nested PCR	Sequence (5'-3')
dlx1a	First	AGGCGGTGGTACGATCGA
	Nested	GTACGATCGACAGCAACGC
dlx2a	First	CTTGCACAAATCGCAGGAGTC
	Nested	CGGGTCACCGTTTGGACA
dlx6a	First	GTCGTTACCTTACCCTTACGT
	Nested	CGGCCTCATCCAAAGGAG
